# New Marine Antifouling Compounds from the Red Alga *Laurencia* sp.

**DOI:** 10.3390/md15090267

**Published:** 2017-08-28

**Authors:** Yuko Oguri, Mami Watanabe, Takafumi Ishikawa, Takashi Kamada, Charles S. Vairappan, Hiroshi Matsuura, Kensuke Kaneko, Takahiro Ishii, Minoru Suzuki, Erina Yoshimura, Yasuyuki Nogata, Tatsufumi Okino

**Affiliations:** 1 Graduate School of Environmental Science, Hokkaido University, Sapporo 060-0810, Japan; oguri-yuu@awi.co.jp (Y.O.); watamatsu@ees.hokudai.ac.jp (M.W.); tishikawa947@gmail.com (T.I.); 2 Institute for Tropical Biology and Conservation, Universiti Malaysia Sabah, Kota Kinabalu, Sabah 88400, Malaysia; takashi.kamada@ums.edu.my (T.K.); csv@mes.edu.my (C.S.V.); 3 National Institute of Technology, Asahikawa College, Asahikawa 071-8142, Japan; matsuura@asahikawa-nct.ac.jp; 4 Graduate School of Pharmaceutical Sciences, Kyoto University, Sakyo-ku, Kyoto 606-8501, Japan; kaneko.kensuke.6a@kyoto-u.ac.jp; 5 Faculty of Agriculture, University of the Ryukyus, Senbaru 1, Nishihara, Okinawa 903-0213, Japan; ishiit@agr.u-ryukyu.ac.jp; 6 Coastal Branch of Natural History Museum and Institute, Chiba, 123 Yoshio, Katsuura, Chiba 299-5242, Japan; reiboku1965@yahoo.co.jp; 7 CERES, Inc., 1-4-5 Midori, Abiko, Chiba 270-1153, Japan; erinamusi@yahoo.co.jp; 8 Environmental Science Research Laboratory, Central Research Institute of Electric Power Industry, 1646 Abiko, Abiko, Chiba 270-1194, Japan; noga@criepi.denken.or.jp; 9 Faculty of Environmental Earth Science, Hokkaido University, Sapporo 060-0810, Japan

**Keywords:** antifouling, biofouling, barnacle, terpenoid, acetogenin, *Laurencia*, Rhodophyta

## Abstract

Six new compounds, omaezol, intricatriol, hachijojimallenes A and B, debromoaplysinal, and 11,12-dihydro-3-hydroxyretinol have been isolated from four collections of *Laurencia* sp. These structures were determined by MS and NMR analyses. Their antifouling activities were evaluated together with eight previously known compounds isolated from the same samples. In particular, omaezol and hachijojimallene A showed potent activities (EC_50_ = 0.15–0.23 µg/mL) against larvae of the barnacle *Amphibalanus amphitrite*.

## 1. Introduction

**Figure 1:**
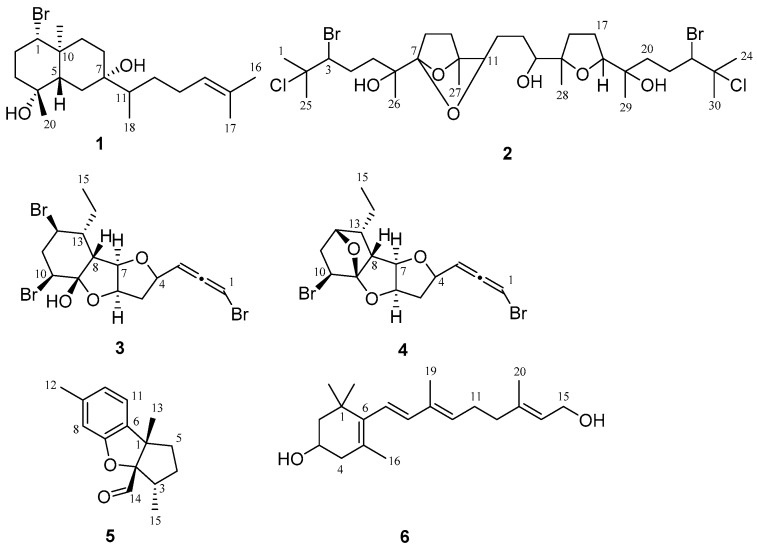
Structures of new compounds isolated from *Laurencia* sp.

Biofouling is a major cause of increased fuel consumption of ships and facilitates the spread of invasive marine organisms [1]. After many years of utilizing toxic antifoulants to control biofouling, the IMO (International Maritime Organization) banned the use of organotin compounds in 2008, paving the way for the development of environmentally friendly fouling-resistant coatings [2]. In addition, effective and new fouling treatment is needed to control and curb the introduction of non-indigenous species (NIS), that are introduced into local waters partly via ballast water uptake and discharge in vessels. The ballast water management convention will enter into force in September 2017. However, to effectively control NIS introduction, an effective antifouling treatment approach is still needed [3]. Search for natural antifouling compounds to solve biofouling has been “a work in progress” for almost four decades. Lately, a number of natural antifouling products have been reported and some of them have shown potent activities [4,5]. Furthermore, recent efforts to identify molecular mechanisms of antifouling compounds improve the possibility of their industrial importance [6]. As expected, industrial utilization of natural products as antifouling agents would warrant systematic synthetic efforts, and these have been implemented [5]. The total synthesis of 10-isocyano-4-cadinene [7] and dolastatin 16 [8] were achieved by our group [9,10]. The red alga *Laurencia* sp. is a rich source of biologically active secondary metabolites [11,12] and produces antifouling compounds such as elatol [13], omaezallene [14], and 2,10-dibromo-3-chloro-7-chamigrene [15]. As a continuous effort, our group has been searching for novel and potent antifouling compounds from *Laurencia* sp. by using barnacle larvae and epiphytic diatom assays. Hence, here we report the structures (Figure 1) and activities of new antifouling compounds from *Laurencia* sp.

## 2. Results

### 2.1. Omaezol (***1***) and Intricatriol (***2***)

**Figure 2:**
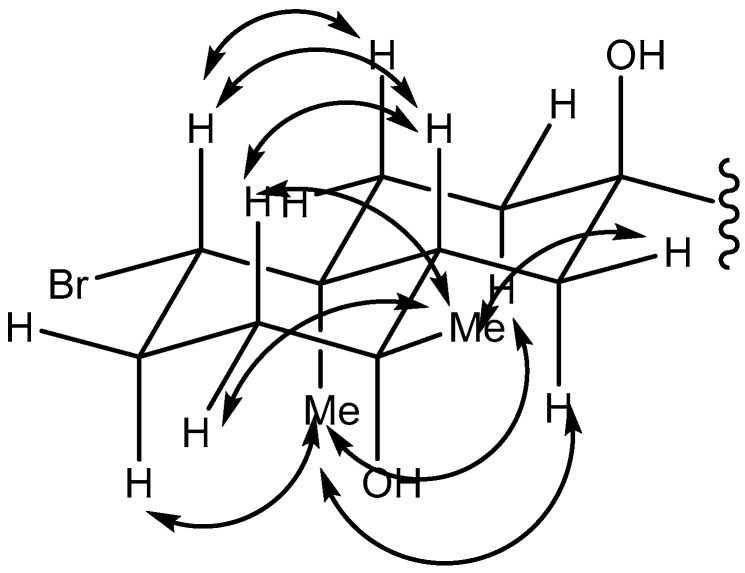
Key NOESY correlations observed in 2D NMR of compound **1**.

*Laurencia* sp., which was collected in Omaezaki, Japan, was extracted with MeOH. The extract yielded omaezol (**1**), intricatriol (**2**), in addition to the previously isolated omaezallenes and intricatetraol [14]. The molecular formula of **1** was determined to be C_20_H_35_BrO_2_ (*m/z* 368.1707, calcd. for C_20_H_33_BrO, 368.1709 [M − H_2_O]^+^) by HR-EIMS (High Resolution-Electron Ionization Mass Spectrometry), suggesting three degrees of unsaturation. The existence of a hydroxy group was revealed by an IR absorption at 3386 cm^−1^. ^13^C NMR data (Table 1) showed the presence of one double bond, suggesting a bicyclic structure. COSY correlations connected C-1 to C-3. HMBC peaks from H-19 to C-1, 5, 9, 10 and from H-20 to C-3, 4, 5 concluded a cyclohexane ring (Figure S1). Based on ^13^C NMR chemical shifts, a bromine atom is attached to C-1 (δ 67.6) and a hydroxy group is connected to C-4 (δ71.4). A side chain was determined by COSY correlation (H-11/H-18 and H-12/H-13) and HMBC peaks (H-18/C-7, 12 and H-14/C-13, 16, 17). Another hydroxy group is attached to C-7 (δ 74.6). Overlapping of NMR chemical shifts (C-6; δ 32.1, C-8; δ 31.8, C-9; δ 38.7, C-10; δ 39.4, H-6; δ 1.48, H-8; δ 1.49) hampered the positioning of the two remaining methylenes. However, HSQC-TOCSY peaks from H-8 to C-9 and H-6 to C-5 concluded the planar structure of **1**. NOESY correlations determined relative configurations of the *trans*-decalin (Figure 2).

The molecular formula of **2** was determined to be C_30_H_52_Br_2_Cl_2_O_6_ (*m/z* 757.1584, calcd. for C_30_H_53_Br_2_Cl_2_O_6_, 757.1581 [M + H]^+^) by HR-FABMS (High Resolution-Fast Atom Bombardment Mass Spectrometry). The ^1^H and ^13^C NMR spectra of **2** (Table 1) were similar to intricatetraol [16,17], but were not symmetric. The 2D NMR interpretations (Figure S2) showed that both compounds had the same carbon skeleton. However, ^13^C chemical shift changes were observed (C-7: δ 84.1 in intricatetraol, δ 113.4 in **2**, and C-11: δ 77.5 in intricatetraol, δ 84.7 in **2**). C-7 in **2** was proposed to be an acetal carbon and C-11 was suggested to be a part of an ether instead of a secondary alcohol to make another five-membered ring. In conclusion, the planar structure of **2** was determined and its configuration was assumed to be the same as intricatetraol [16,17], which was extracted from the same sample.

### 2.2. Hachijojimallenes A (***3***) and B (***4***)

Hachijojimallenes A (**3**) and B (**4**) were isolated together with *N*-methyl-2,3,6-tribromoindole [18] and pinnaterpene C [19] from a red alga, *Laurencia* sp., which was collected in Hachijojima Island, Japan. Based on HR-EIMS data, the molecular formula of **3** was determined to be C_15_H_19_Br_3_O_3_ (*m*/*z* 466.8851, calcd. for C_15_H_18_Br_3_O_2_, 466.8852 [M − OH]^+^). The existence of a bromoallene moiety was suggested by an IR absorption at 1957 cm^−1^, ^13^C NMR chemical shifts (δ 74.1, 99.4, and 201.9), and ^1^H NMR chemical shifts (δ 5.45 and 6.08) (Table 2). COSY correlations established a carbon skeleton C-3—C-8—C-13—C-10 and an ethyl group. ^13^C chemical shifts revealed that C-4, 6, and 7 were oxymethines and C-10 and 12 were brominated. These carbons and an acetal carbon (δ 102.1) were assembled by HMBC data to conclude the planar structure of **3** (Figure S3). Relative configurations were determined by NOEs (H-6/H-10, H-10/H-11 α, H-11 α/H-12, H-7/H-12, and H-8/H-13).

The molecular formula of **4** was determined to be C_15_H_18_Br_2_O_3_ (*m*/*z* 403.9627, calcd. for C_15_H_18_Br_2_O_3_, 403.9617 [M]^+^) by HR-EIMS, suggesting the existence of another ring. The striking difference in its ^13^C NMR (Table 2) was observed in C-9 (**3**; δ 102.1, **4**; δ 119.7) and C-12 (**3**; δ 51.3, **4**; δ 75.6). In addition, HMBC was observed from H-12 to C-9. In conclusion, the hemiacetal in **3** was replaced by acetal in **4** to form an ether between C-9 and C-12, which was debrominated at C-12. NOEs of **4** supported its relative configurations, similar to those of **3**.

### 2.3. Debromoaplysinal (***5***)

Re-investigation of the red alga *L. okamurae*, collected at Oshoro Bay, Hokkaido, Japan, yielded debromoaplysinal (**5**). The molecular formula of **5** was determined to be C_15_H_18_O_2_ (*m*/*z* 231.1379, calcd. for C_15_H_19_O_2_, 231.1380 [M + H]^+^) by ESI-TOFMS. Its 1D NMR spectra (Table 2) revealed the existence of an aldehyde and a trisubstituted benzene. COSY and HMBC correlations (Figure S4) clarified its planar structure, which is similar to aplysinal and debromoaplysinol. In fact, chemical shifts in the trisubstituted benzene of **5** are similar to those of debromoaplysinol, and those in the cyclopentane of **5** are similar to those of aplysinal. NOESY peaks between H-13 and H-14 suggested *cis* configuration of Me-13 and the aldehyde. Irradiation of H-3 by DPFGSE 1D NOE showed the enhancement of H-13. In conclusion, H-3, Me-13, and the aldehyde are located on the same face.

### 2.4. 11,12-Dihydro-3-hydroxyretinol (***6***)

Re-investigation of the red alga *L. nipponica* collected at Muroran, Hokkaido, Japan, yielded 11,12-dihydro-3-hydroxyretinol (**6**), along with three known halogenated chamigrene-type sesquiterpenoids [20,21]. The molecular formula of **6** was deduced to be C_20_H_32_O_2_ (*m*/*z* 304.2400, calcd. for C_20_H_32_O_2_, 304.2397 [M]^+^) by HR-EIMS, accounting for five degrees of unsaturation. The ^1^H- and ^13^C-NMR spectroscopic data (Table 3) as well as the HSQC experiment of **6** showed the presence of eight sp^2^ carbons, three vinyl methyls, a pair of geminal methyls, an oxymethylene, an oxymethine, four methylenes, and a quaternary carbon. These signals explained four degrees of unsaturation, implying that one ring was present in **6**. COSY and HMBC data (Figure S5) clearly indicated the gross structure of **6**. The *E* configurations of double bonds were deduced from the ^1^H-^1^H coupling constants (^3^*J*_7–8_ 15.9 Hz) and ^13^C-NMR chemical shifts of vinyl methyls (C-19; δ 13.2, and C-20; δ 17.2). Unfortunately, the configuration at C-3 was not determined due to its limited amount.

### 2.5. Antifouling Activity

**Figure 3:**
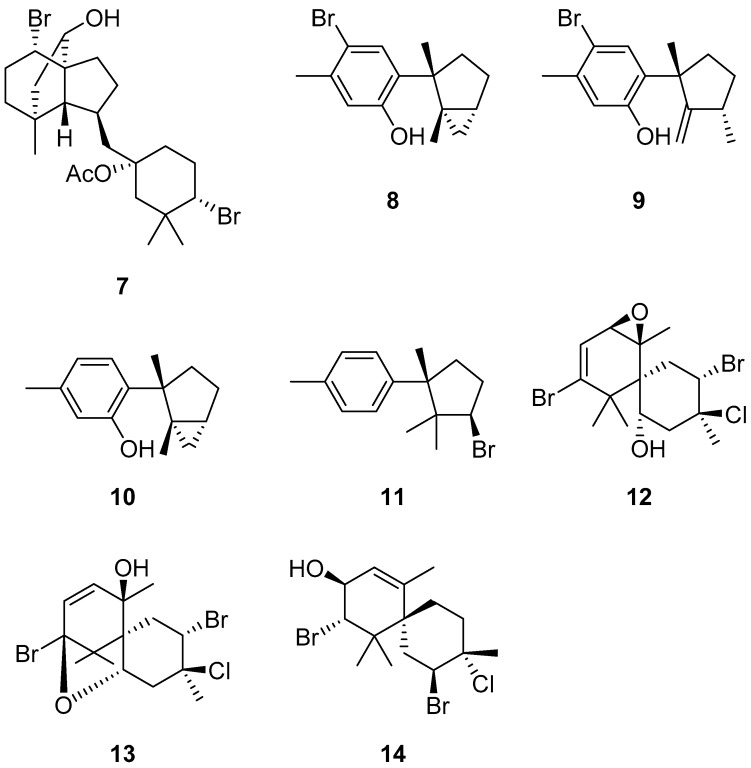
Structures of tested known compounds isolated from *Laurencia* sp.

Most of the compounds obtained in this study were tested for antifouling activity against larvae of the barnacle *Amphibalanus amphitrite* (Table 4). The antifouling activity of **1** was potent (EC_50_ = 0.23 µg/mL) and its toxicity was low (LC_50_ = 3.7 µg/mL), while **2** was not potent (EC_50_ > 10 µg/mL). The antifouling activity of **3** was potent (EC_50_ = 0.15 µg/mL) and its toxicity was low (LC_50_ = 9.8 µg/mL), while **4** was a little less potent (EC_50_ = 0.31 µg/mL, LC_50_ = 6.8 µg/mL). The antifouling activity of the known compound, pinnaterpene C (**7**, EC_50_ = 0.82 µg/mL, LC_50_ > 10 µg/mL), was similar to that of **4**, while another known compound, *N*-methyl-2,3,6-tribromoindole, showed weak activity (EC_50_ = 4.3 µg/mL, not toxic at 10 µg/mL). Compound **5** also showed moderate antifouling activity (EC_50_ = 1.0 µg/mL) and did not kill any larvae at 10 µg/mL. Some known compounds (Figure 3) isolated from *L. okamurae* were tested. Three aromatic sesquiterpenoids [22] showed antifouling activities (laurinterol **8**: EC_50_ = 0.65 µg/mL, LC_50_ = 5.8 µg/mL; isolaurinterol **9**: EC_50_ = 0.34 µg/mL, LC_50_ > 10 µg/mL; debromolaurinterol **10**: EC_50_ = 0.5 µg/mL, LC_50_ > 10 µg/mL), while α-bromocuparene (**11**) was inactive. Three sesquiterpenoids (Figure 3) isolated from *L. nipponica* collected in Muroran showed antifouling activities (prepacifenol **12**: EC_50_ = 0.63 µg/mL, LC_50_ > 10 µg/mL; pacifenol **13**: EC_50_ = 2.36 µg/mL, LC_50_ > 10 µg/mL; 2,10-dibromo-3-chloro-9-hydroxy-α-chamigrene **14** EC_50_ = 2.51 µg/mL, LC_50_ > 10 µg/mL). In addition, anti-diatom activities against the epiphytic marine diatoms, *Nitzschia* sp. and *Cylindrotheca closterium*, were tested for three chamigrenes and **6**. Compounds **6**, **12**, and **14** showed inhibition against both diatoms at 5–10 µg/cm^2^.

## 3. Discussion

From a chemical point of view, **1** is a rare halo-diterpenoid, prenylated selinene-type compound like anhydroaplysiadiol [23]. Oxasqualenoids such as **2** have been proposed to be synthesized by the epoxide-opening cascades. These reactions could be catalyzed by vanadium-dependent bromoperoxidases [24]. However, we do not know how different cyclization patterns are controlled to produce **2** and the similar compound intricatetraol [16,17]. Although most of C_15_ acetogenins from *Laurencia* contain only ether rings, **3** and **4** contain a carbocyclic ring, similar to lembynes [25]. Compound **5** is one of common laurane-type sesquiterpenes. Retinols occur in nature only in animals and in the limited green algae *Caulerpa* sp. [26] This is the first record of a retinane-type diterpene (**6**) isolated from *Laurencia*. Although the genus of *Laurencia* is one of the most studied organisms in marine natural product chemistry [11,12], recent isolation techniques yielded six new compounds in this study. Twelve compounds showed antifouling activities against the barnacle larvae. Notably, the activities of **1** and **3** were equivalent to CuSO_4_ (EC_50_ = 0.18 µg/mL). Three compounds showed antifouling activities against diatoms. However, most of compounds isolated from *Laurencia* sp. so far have not been tested for antifouling activity. Our results suggest that *Laurencia* is a potential source of antifouling compounds.

## 4. Materials and Methods

### 4.1. General Procedures

IR spectra were measured on a JASCO IR-700 spectrophotometer (JASCO, Tokyo, Japan). ^1^H NMR and ^13^C NMR spectra were recorded in CDCl_3_ by using JEOL JNM-ECA600 (JEOL, Tokyo, Japan), JEOL JNM-EX400 (JEOL, Tokyo, Japan), or BRUKER ASX300 spectrometer (Bruker BioSpin, Faellanden, Switzerland), unless otherwise stated. EI-MS were obtained on a JEOL JMS-FABmate spectrometer (JEOL, Tokyo, Japan). FAB-MS were obtained on a JEOL JMS-HX110 spectrometer (JEOL, Tokyo, Japan). ESI-MS were obtained on a JEOL JMS-700TZ (JEOL, Tokyo, Japan) or BRUKER DALTONICS micro TOF-HS focus spectrometer (Bruker Daltonics, Bremen, Germany). Optical rotations were recorded on a HORIBA SEPA-300 polarimeter (Horiba, Kyoto, Japan).

### 4.2. Plant Material

Algal samples of *Laurencia* sp. were collected at Omaezaki, Shizuoka Prefecture, and Hachijojima Island, Tokyo, Japan. *L. okamurae* was collected at Oshoro Bay and *L. nipponica* was collected at Muroran, Hokkaido, Japan. The voucher specimens are deposited in the Herbarium of Graduate School of Science, Hokkaido University.

### 4.3. Omaezol and Intricatriol

The dried algal sample (250 g) was extracted and separated as described in a previous paper [12]. Omaezol (**1**, 3.2 mg) and intricatriol (**2**, 17.0 mg) were isolated by HPLC (YMC-Pack Pro C18 (YMC, Kyoto, Japan) with CH_3_CN and H_2_O) from the omaezallene containing silica-gel fraction.

**1**: ${\left[\alpha \right]}_{D}^{23}$ −67.4 (*c* 0.17, CHCl_3_); IR (neat), ν_max_ 3428, 1259, 1185, 1088, 1065, 954, 801 cm^−1^; ^1^H NMR and ^13^C NMR, see Table 1.

**2**: ${\left[\alpha \right]}_{D}^{23}$ −45.2 (*c* 0.39, CHCl_3_) ; IR (neat), ν_max_ 3414, 2972, 1451, 1369, 1214, 1101, 756 cm^−1^; ^1^H NMR and ^13^C NMR, see Table 1.

### 4.4. Hachijojimallenes A and B

The air-dried algae (80 g) were soaked in MeOH (500 mL) for seven days. The MeOH solution was concentrated in vacuo, and the residue was partitioned between EtOAc and H_2_O. The EtOAc layer was then washed with water, dried over anhydrous Na_2_SO_4_, and evaporated in vacuo to leave a dark green oily substance (1.23 g). The extract (700 mg) was fractionated by Si gel CC with a step gradient (hexane and EtOAc). The fraction (41 mg) eluted with hexane-EtOAc (9:1) was further subjected to PTLC (preparative thin-layer chromatography) with hexane to give 1-methyl-2,3,6-tribromoindole (4.0 mg), which was determined on the basis of ^1^H NMR and MS data. The fraction (411 mg) eluted with hexane–EtOAc (7:3) was further chromatographed on a Si gel column with hexane–EtOAc (3:1) (each eluate, 10 mL) to give 10 fractions. The fourth fraction (79.1 mg) was then separated by PTLC with hexane-EtOAc (3:1) to afford hachijojimallene B (**4**) (3.5 mg). The combined fifth and sixth fractions (74.9 mg) were further separated by repeated PTLC with hexane–EtOAc (1:1) to give hachijojimallene A (**3**) (40.0 mg). The seventh fraction (43.8 mg) was subjected to PTLC with hexane–EtOAc (3:1) followed by HPLC (Develosil-ODS-T-5 with CH_3_CN and H_2_O) to give pinnaterpene C (**7**, 15.0 mg).

**3**: ${\left[\alpha \right]}_{D}^{24}$ −88.2 (*c* 0.13, CHCl_3_); IR (neat), ν_max_ 3490, 1957, 1258, 1184, 1165, 1143, 1120, 1038, 959, 788, 756 cm^−1^; ^1^H NMR and ^13^C NMR, see Table 1.

**4**: ${\left[\alpha \right]}_{D}^{24}$ −151 (*c* 0.71, CHCl_3_); IR (neat), ν_max_ 1950, 1243, 1181, 1127, 1080, 1045, 1022, 970, 855, 821 cm^−1^; ^1^H NMR and ^13^C NMR, see Table 1.

### 4.5. Debromoaplysinal

The air-dried algae (100 g) were soaked in MeOH (3 L) for seven days. The MeOH solution was concentrated in vacuo, and the residue was partitioned between EtOAc and H_2_O. The EtOAc layer was then washed with water, dried over anhydrous Na_2_SO_4_, and evaporated in vacuo to obtain the crude extract (4.3 g). The extract (2.1 g) was fractionated by Si gel column chromatography with a step gradient (hexane and EtOAc). The fraction (1.4 g) eluted with hexane-EtOAc (3:1) was further subjected to two sets of Si gel column chromatography and PTLC with toluene to give debromoaplysinal (**5**) (0.9 mg). The other known compounds (laurinterol **8**, 30.6 mg; isolaurinterol **9**, 12.4 mg; debromolaurinterol **10**, 11.0 mg; α-bromocuparene **11**, 1.1 mg) were isolated from the same fraction (hexane–EtOAc 3:1) in a similar way.

**5**: ${\left[\alpha \right]}_{D}^{24}$ −91.9 (*c* 0.08, CHCl_3_); IR (neat), ν_max_ 2927, 2856, 1731, 1593, 1499, 1456, 1377, 1266, 1146, 1117, 971, 805 cm^−1^; ^1^H NMR and ^13^C NMR, see Table 1.

### 4.6. 11,12-Dihydro-3-hydroxyretinol

Fresh algal sample (400 g wet weight) was extracted in MeOH at room temperature for three days. The resulting MeOH extract was concentrated in vacuo and partitioned between diethyl ether and H_2_O. The diethyl ether fraction (340 mg) was subjected to Si gel CC eluting with a gradient of hexane and EtOAc in an increasing polarity. Fraction 4, eluted with hexane-EtOAc (3:1), was subjected to PTLC to yield pacifenol (**12**, 4.1 mg) and prepacifenol (**13**, 6.3 mg). Fraction 5, eluted with hexane-EtOAc (1:1), was subjected to PTLC with CHCl_3_-MeOH (97:3) to yield 2,10-dibromo-3-chloro-9-hydroxy-α-chamigrene (**14**, 7.3 mg). Fraction 7, eluted with hexane-EtOAc (1:3), gave **6** (1.3 mg) after purification with PTLC using CHCl_3_-MeOH (95:5).

**6**: ${\left[\alpha \right]}_{D}^{27}$ −61.9 (*c* 0.07, CHCl_3_); ^1^H NMR and ^13^C NMR, see Table 1.

### 4.7. Antifouling Assay

An antifouling assay against larvae of the barnacle *Amphibalanus amphitrite* was conducted according to the previous literature [9]. The antifouling assay against diatoms *Nitzschia* sp. and *Cylindrotheca closterium* was conducted as follows. The diatoms were maintained in test tubes under 16 h light and 8 h dark cycle conditions at 25 °C in modified Jorgensen’s medium. Pure compounds were applied to a sample zone (16 mm in diameter) of cellulose TLC aluminum sheet (58 × 58 mm), and then each treated sheet was placed in Petri dish (15 × 90 mm in diameter). After that, 25-mL aliquots of modified Jorgensen’s medium were introduced into Petri dishes with treated sheets, and inoculated with 1 mL of cultivated diatom suspension in equivalent cell densities. Petri dishes were sealed with Parafilm to ensure a closed system, and were incubated under the same conditions. Cell growth was estimated daily, and the adhesive condition was evaluated and compared to those of the control after seven days of incubation.

## Figures and Tables

**Table 1:** ^1^H and ^13^C NMR spectroscopic data for compounds **1** and **2** in CDCl_3_.

Carbon Number	Compound 1		Compound 2		Carbon Number	Compound 2	
	^13^C	^1^H	^13^C	^1^H		^13^C	^1^H
**1**	67.6	3.91	27.8	1.689	**24**	27.5	1.695
**2**	30.2	2.44, 2.01	72.0		**23**	72.0	
**3**	41.9	1.55, 1.70	67.1	4.07	**22**	67.1	4.08
**4**	71.4		28.7	1.85, 2.61	**21**	28.9	1.75, 2.45
**5**	48.2	1.10	36.8	1.72, 1.89	**20**	36.9	1.47, 1.72
**6**	32.1	1.48, 2.00	72.2		**19**	73.3	
**7**	74.6		113.4		**18**	84.3	3.85
**8**	31.8	1.49, 1.90	27.9	1.60, 1.70	**17**	26.4	1.93
**9**	38.7	1.05, 1.76	32.8	1.91	**16**	32.7	1.57, 2.19
**10**	39.4		86.7		**15**	85.6	
**11**	33.6	1.67	84.7	3.61	**14**	76.0	3.57
**12**	30.2	1.52	29.6	1.55, 2.40	**13**	29.4	1.74
**13**	26.5	1.12			**25**	33.1	1.785
**14**	124.3	5.10			**30**	32.9	1.793
**15**	131.7				**26**	21.2	1.28
**16**	25.5	1.70			**29**	24.5	1.26
**17**	17.6	1.62			**27**	17.6	1.45
**18**	12.3	0.90			**28**	23.0	1.19
**19**	14.4	1.26					
**20**	29.7	1.18					

**Table 2:** ^1^H and ^13^C NMR spectroscopic data for compounds **3**–**5** in CDCl_3_.

Carbon Number	Compound 3		Compound 4		Compound 5	
	^13^C	^1^H	^13^C	^1^H	^13^C	^1^H
**1**	74.1	6.08	74.0	3.09	58.7	
**2**	201.9		201.9		103.4	
**3**	99.4	5.45	100.0	5.48	42.4	2.59
**4**	73.8	4.73	74.1	4.72	31.7	1.26, 1.74
**5**	39.3	1.81, 2.26	39.6	1.82, 2.35	43.0	1.74, 1.92
**6**	79.4	4.56	88.6	5.11	131.6	
**7**	81.4	4.61	82.8	4.77	159.4	
**8**	50.5	2.77	51.0	3.19	110.0	6.72
**9**	102.1		119.7		138.8	
**10**	53.6	4.07	42.8	4.13	122.1	6.74
**11**	44.3	2.50, 2.76	40.6	1.74, 2.70	122.4	6.92
**12**	51.3	3.79	75.6	4.03	21.5	2.33
**13**	44.7	1.97	50.2	2.13	24.2	1.31
**14**	24.7	1.47, 2.11	23.3	1.44, 1.60	203.5	9.78
**15**	11.6	1.03	12.5	0.99	13.1	1.04
9-OH		3.65				

**Table 3:** ^1^H and ^13^C NMR spectroscopic data for compound **6** in CDCl_3_.

Carbon Number	^13^C	^1^H	Carbon Number	^13^C	^1^H
**1**	37.9		**11**	27.5	2.29
**2**	49.1	1.47, 1.76	**12**	40.0	2.12
**3**	65.9	4.00	**13**	140.0	
**4**	43.2	2.02, 2.37	**14**	124.2	5.44
**5**	125.9		**15**	60.1	4.18
**6**	138.2		**16**	22.4	1.71
**7**	124.0	5.92	**17**	31.0	1.06
**8**	139.0	6.00	**18**	29.5	1.06
**9**	134.6		**19**	13.2	1.79
**10**	131.4	5.40	**20**	17.2	1.71

**Table 4:** Antifouling activities (µM) against barnacle larvae.

Compound	EC_50_	LC_50_
**1**	0.59	9.6
**2**	—	—
**3**	0.31	20
**4**	0.76	17
**5**	4.3	—
**7**	1.6	—
**8**	2.2	20
**9**	1.2	—
**10**	2.3	—
**11**	—	—
**12**	1.5	—
**13**	5.5	—
**14**	6.1	—
CuSO_4_	0.72	1.4

—: EC_50_ or LC_50_ is more than 10 µg/mL.

## Supplementary Materials

The following are available online at www.mdpi.com/1660-3397/15/9/267/s1, Figure S1: Key 2D NMR correlations of compound **1**; Figure S2: Key 2D NMR correlations of compound **2**; Figure S3: Key 2D NMR correlations of compound **3**; Figure S4: Key 2D NMR correlations of compound **5**; Figure S5: Key 2D NMR correlations of compound **6**; Figure S6: ^1^H NMR spectrum of omaezol (1) in CDCl_3_; Figure S7: ^13^C NMR spectrum of omaezol (1) in CDCl_3_; Figure S8: ^1^H NMR spectrum of intricatriol (2) in CDCl_3_; Figure S9: ^13^C NMR spectrum of intricatriol (2) in CDCl_3_; Figure S10: ^1^H–^1^H COSY spectrum of intricatriol (2) in CDCl_3_; Figure S11: HSQC spectrum of intricatriol (2) in CDCl_3_; Figure S12: HMBC spectrum of intricatriol (2) in CDCl_3_; Figure S13: NOESY spectrum of intricatriol (2) in CDCl_3_; Figure S14: ^1^H NMR spectrum of hachijojimallene A (3) in CDCl_3_; Figure S15: ^13^C NMR spectrum of hachijojimallene A (3) in CDCl_3_; Figure S16: ^1^H–^1^H COSY spectrum of hachijojimallene A (3) in CDCl_3_; Figure S17: HMQC spectrum of hachijojimallene A (3) in CDCl_3_; Figure S18: HMBC spectrum of hachijojimallene A (3) in CDCl_3_. Figure S19: NOESY spectrum of hachijojimallene A (3) in CDCl_3_; Figure S20: ^1^H NMR spectrum of hachijojimallene B (4) in CDCl_3_; Figure S21: ^13^C NMR spectrum of hachijojimallene B (4) in CDCl_3_. Figure S22. HMQC spectrum of hachijojimallene B (4) in CDCl_3_; Figure S23: HMBC spectrum of hachijojimallene B (4) in CDCl_3_; Figure S24: ^1^H NMR spectrum of debromoaplysinal (5) in CDCl_3_; Figure S25: ^13^C NMR spectrum of debromoaplysinal (5) in CDCl_3_; Figure S26: DPFGSE 1D NOE spectrum of debromoaplysinal (5) in CDCl_3_; Figure S27: ^1^H NMR spectrum of 11,12-dihydro-3-hydroxyretinol (6) in CDCl_3_; Figure S28: ^13^C NMR spectrum of 11,12-dihydro-3-hydroxyretinol (6) in CDCl_3_.
