# Supplementary material for: New Marine Antifouling Compounds from the Red Alga Laurencia sp
Source: Mar Drugs. 2017 Aug 28;15(9):267. doi: 10.3390/md15090267 (PMC5618406; doi:10.3390/md15090267)
Supplement: Supplementary file 1 [file marinedrugs-15-00267-s001.pdf]

Supplementary Materials

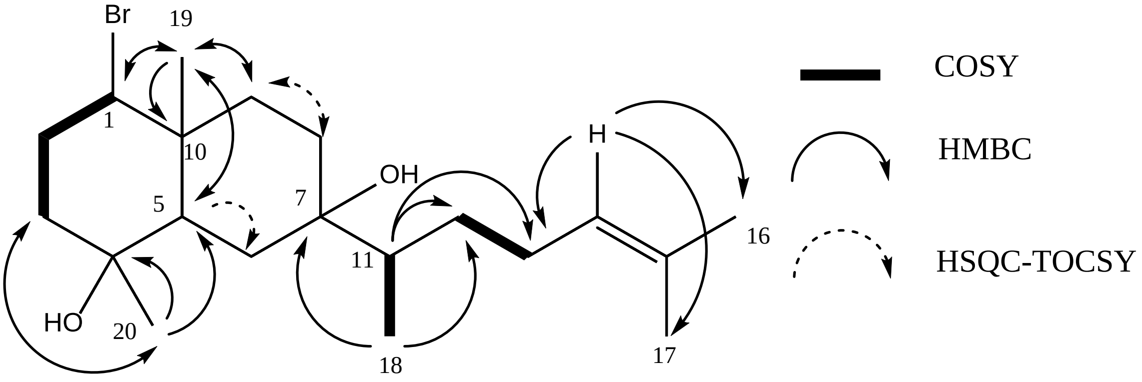

Figure S1. Key 2D NMR correlations of compound **1**.

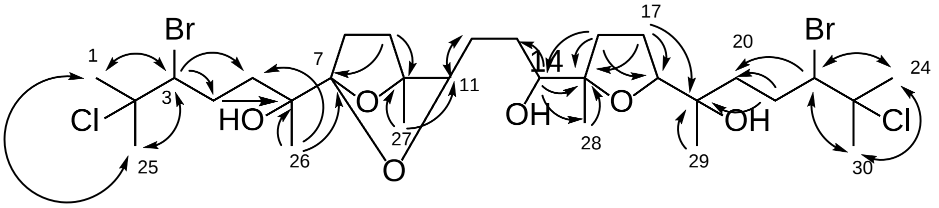

Figure S2. Key 2D NMR correlations of compound **2**.

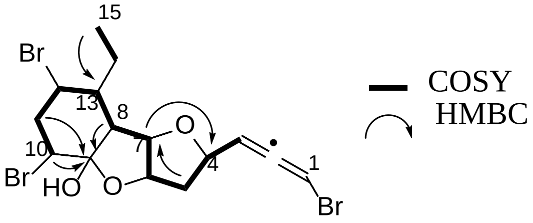

Figure S3. Key 2D NMR correlations of compound **3**.

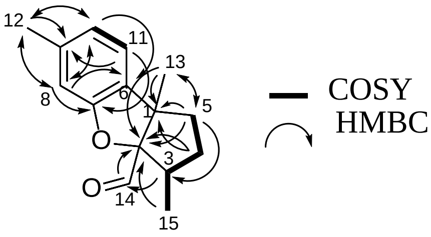

Figure S4. Key 2D NMR correlations of compound **5**.

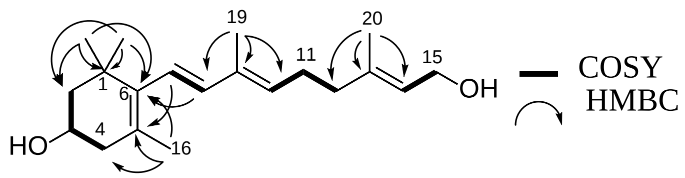

Figure S5. Key 2D NMR correlations of compound **6**.

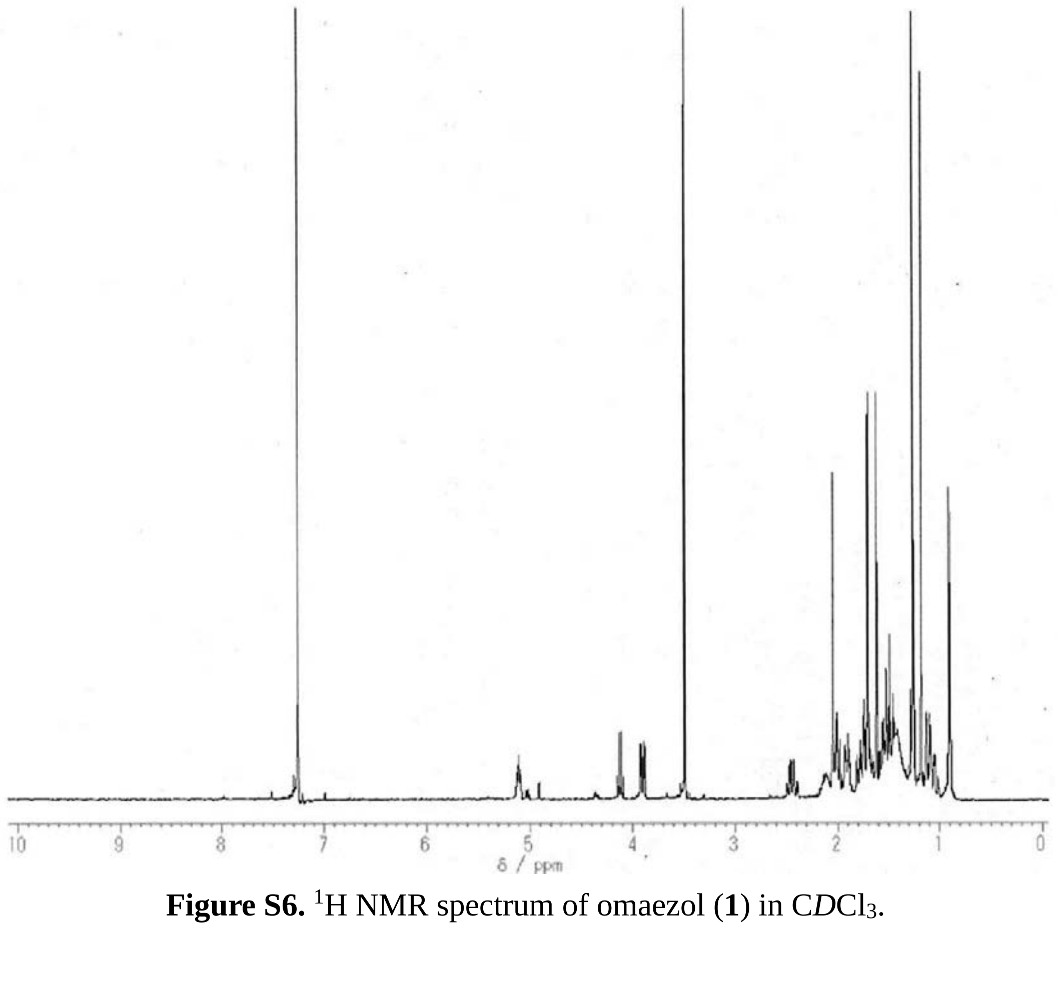

Figure S6. ^{1}H NMR spectrum of omaezol (**1**) in C*D*Cl$_3$.

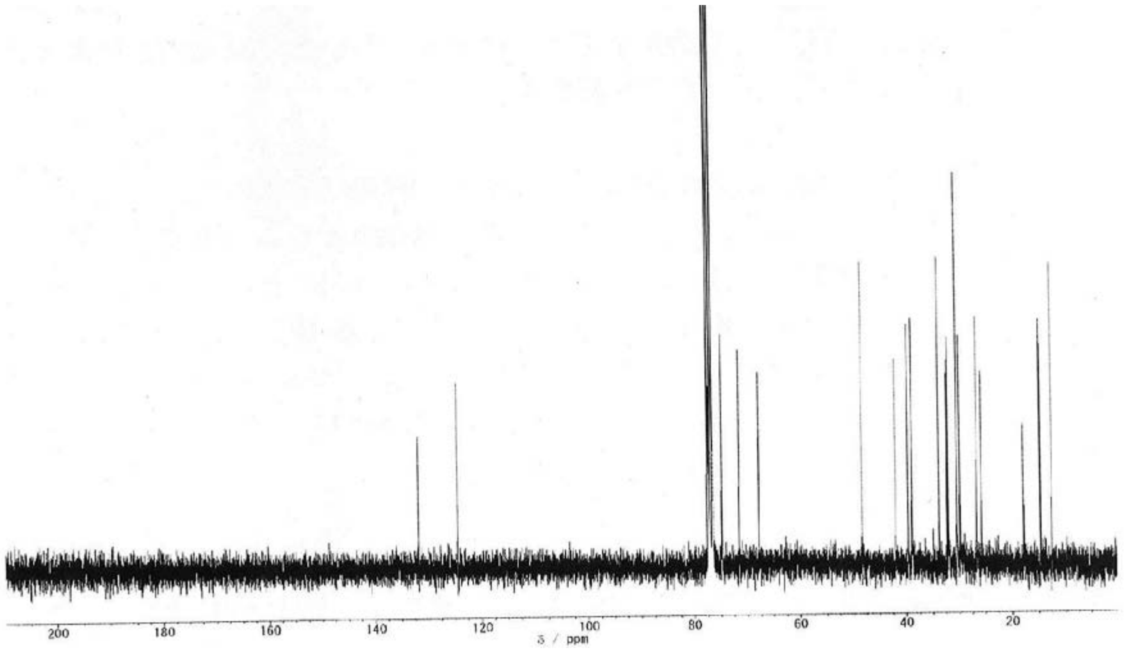

Figure S7. ^{13}C NMR spectrum of omaezol (**1**) in C*D*Cl_3.

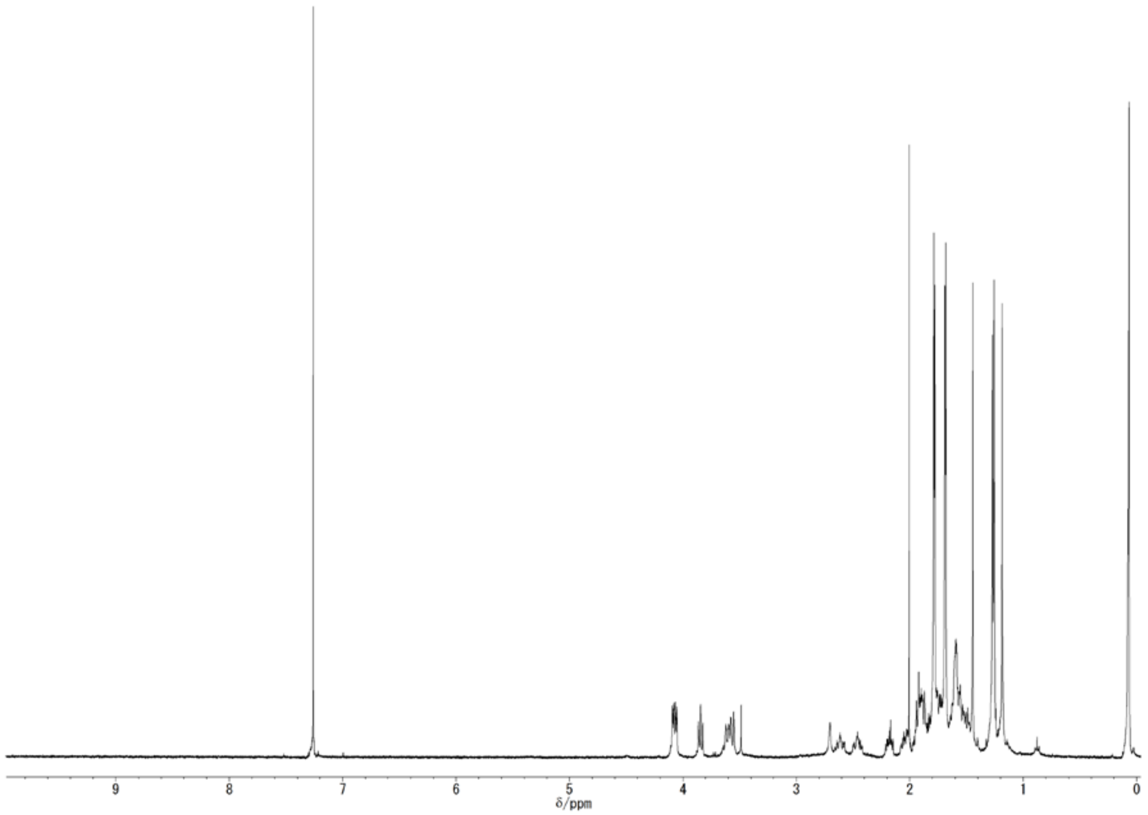

Figure S8. ^{1}H NMR spectrum of intricatriol (**2**) in C*D*Cl_3.

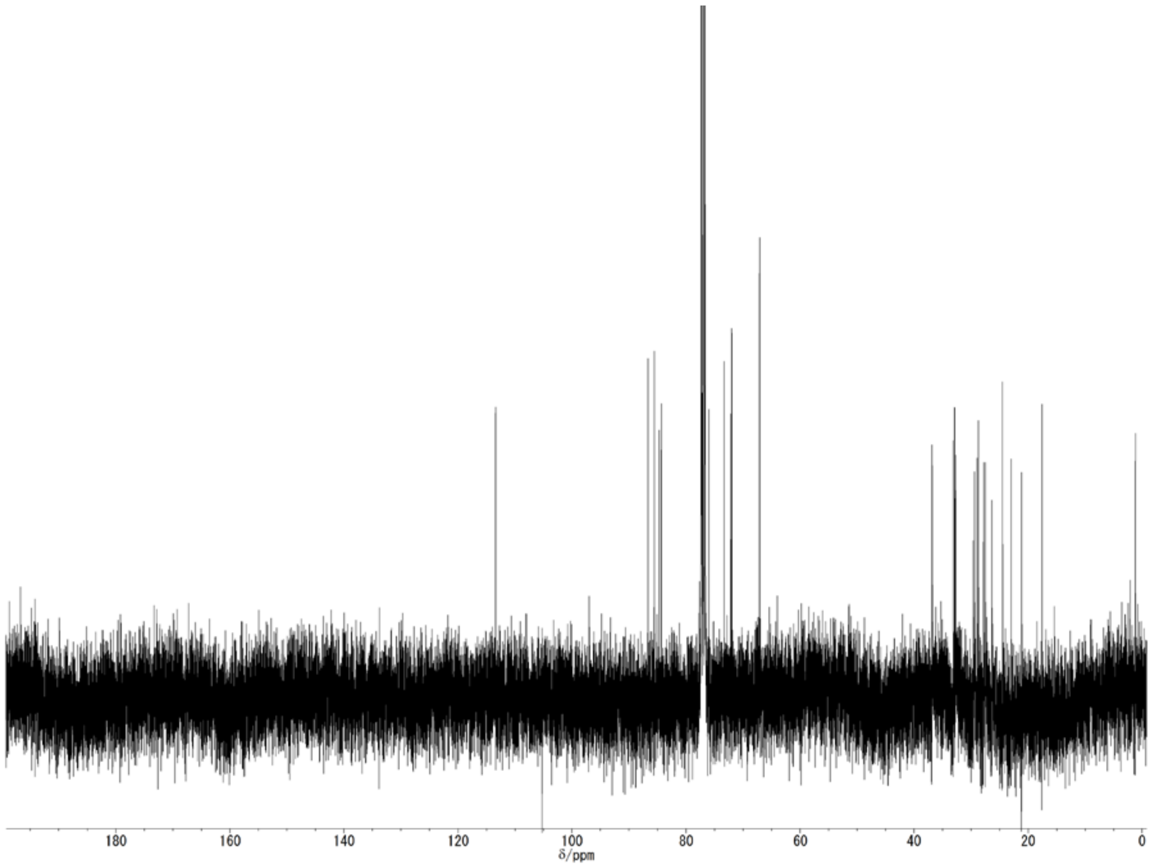

Figure S9. ^{13}C NMR spectrum of intricatriol (**2**) in C*D*Cl_3.

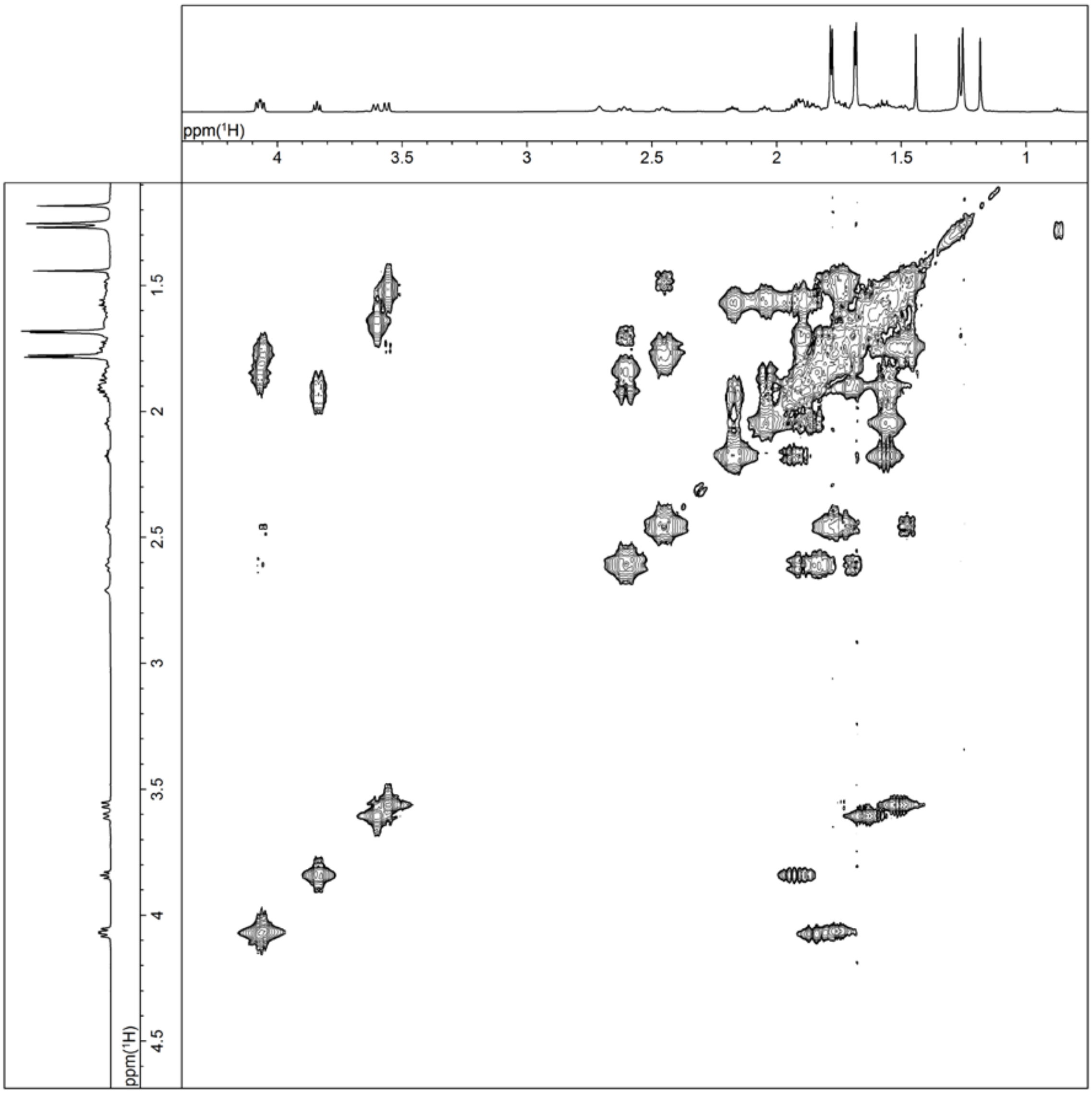

Figure S10. ^{1}H–^{1}H COSY spectrum of intricatriol (**2**) in C*D*Cl_3.

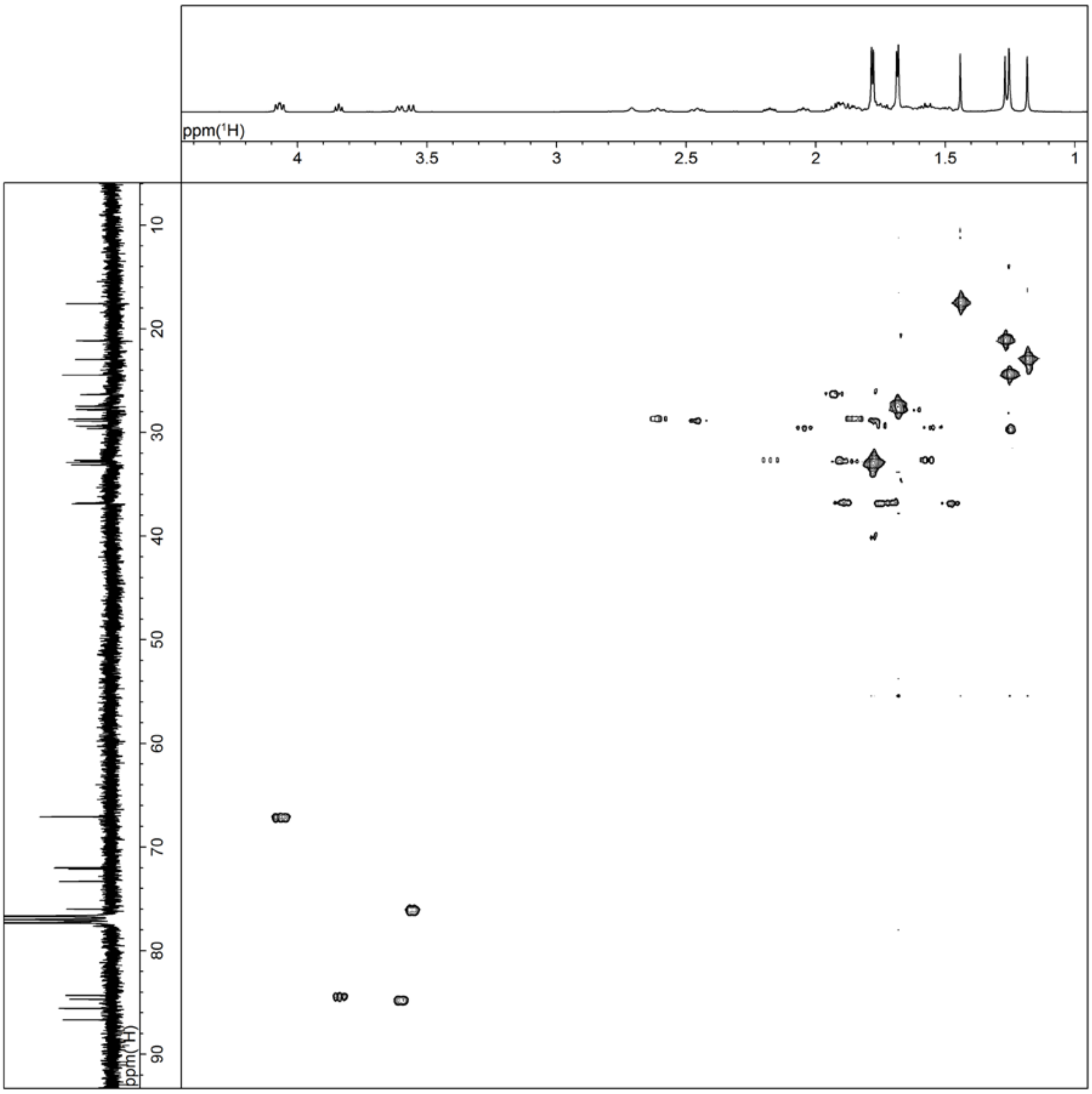

Figure S11. HSQC spectrum of intricatriol (**2**) in C*D*Cl$_3$.

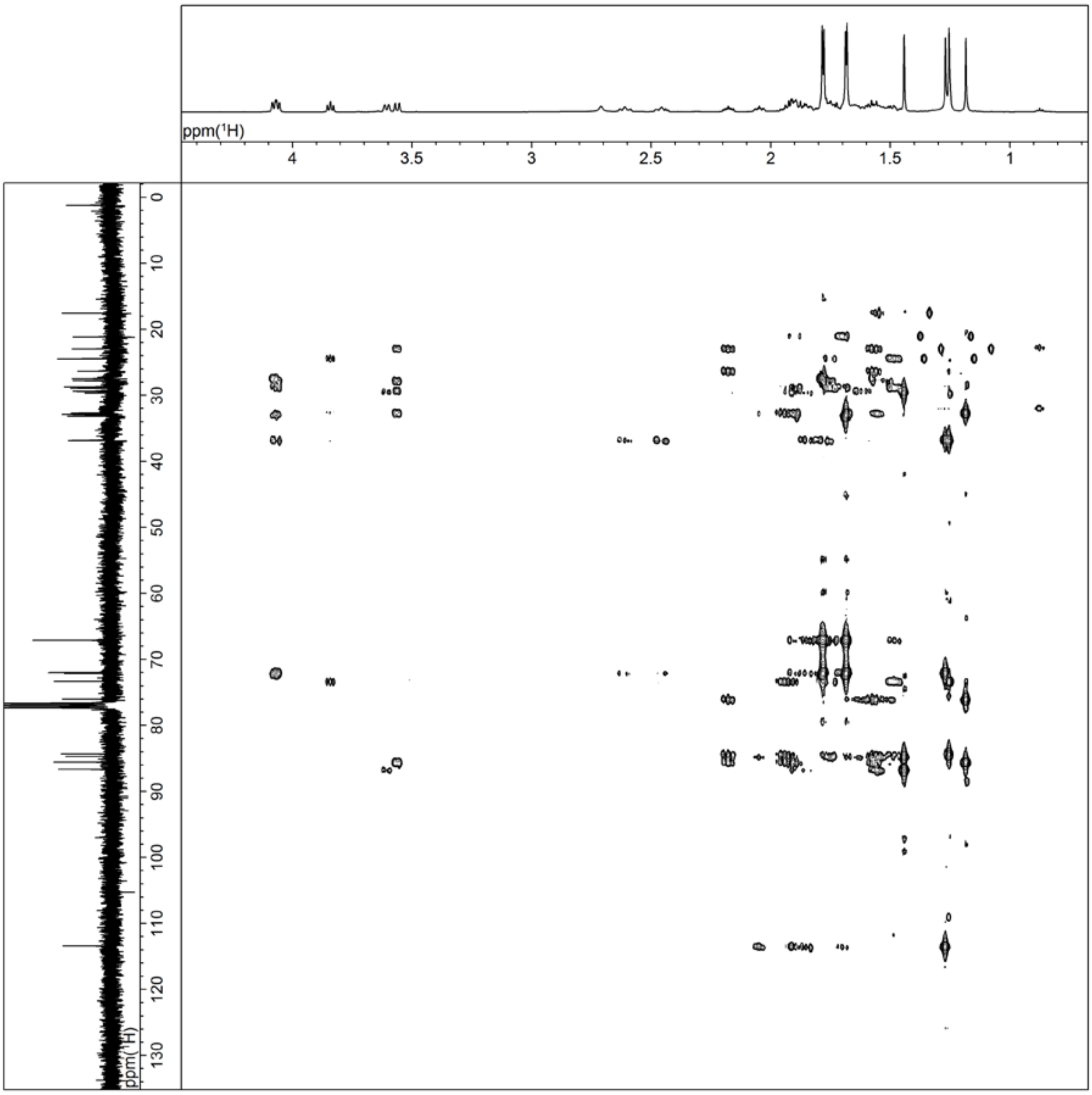

Figure S12. HMBC spectrum of intricatriol (**2**) in C*D*Cl$_3$.

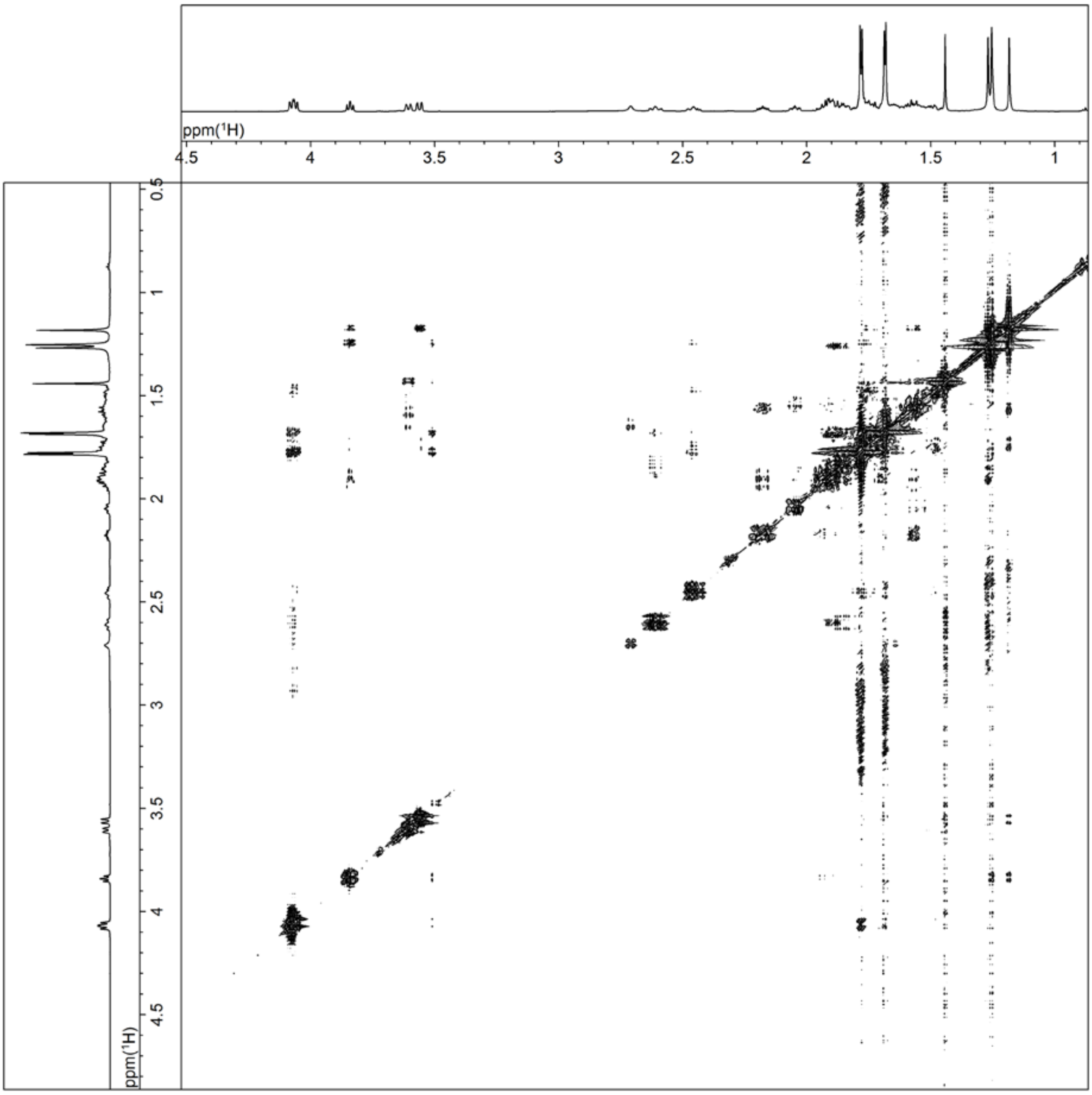

Figure S13. NOESY spectrum of intricatriol (**2**) in C*D*Cl_3.

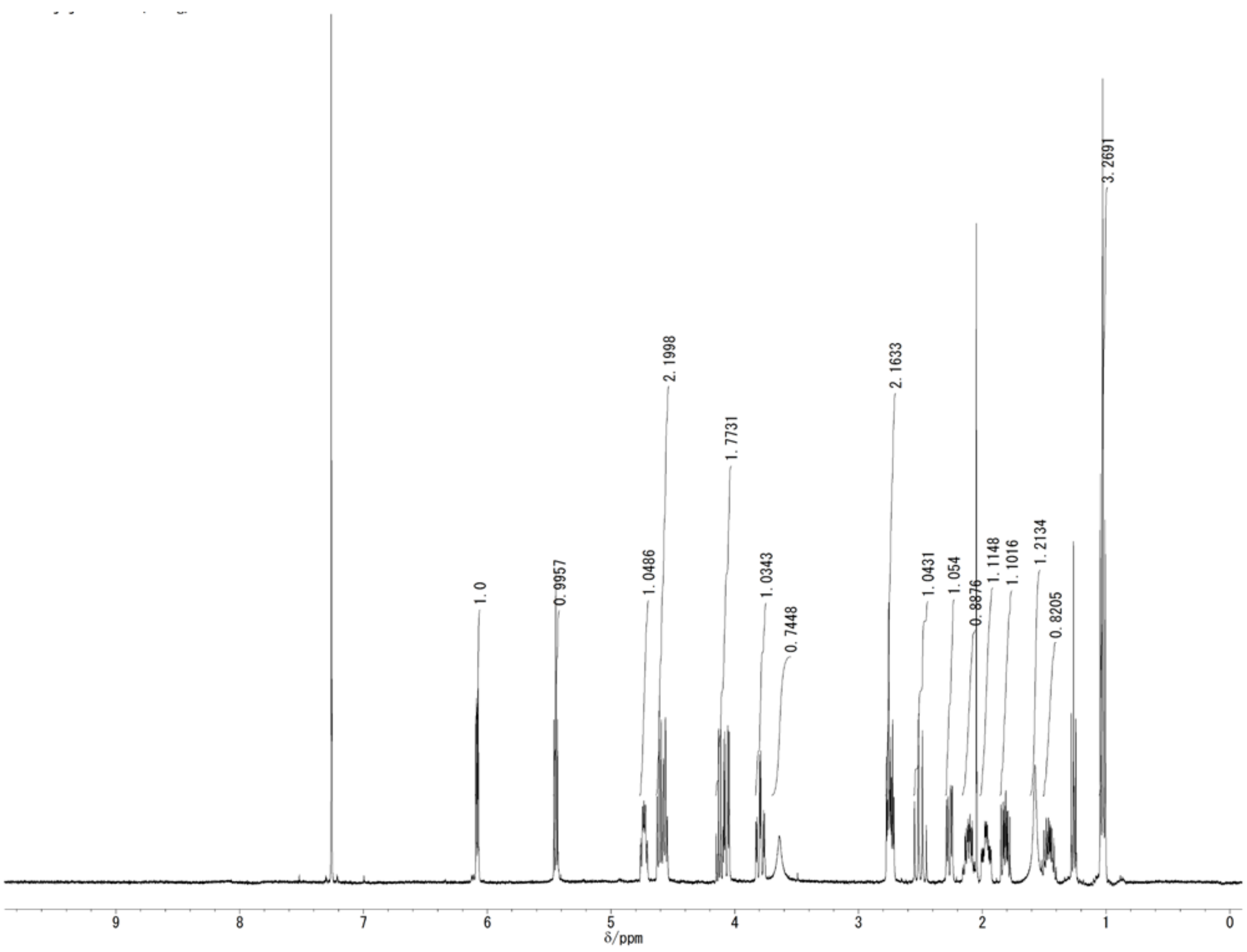

Figure S14. ^{1}H NMR spectrum of hachijojimallene A (**3**) in C*D*Cl$_3$.

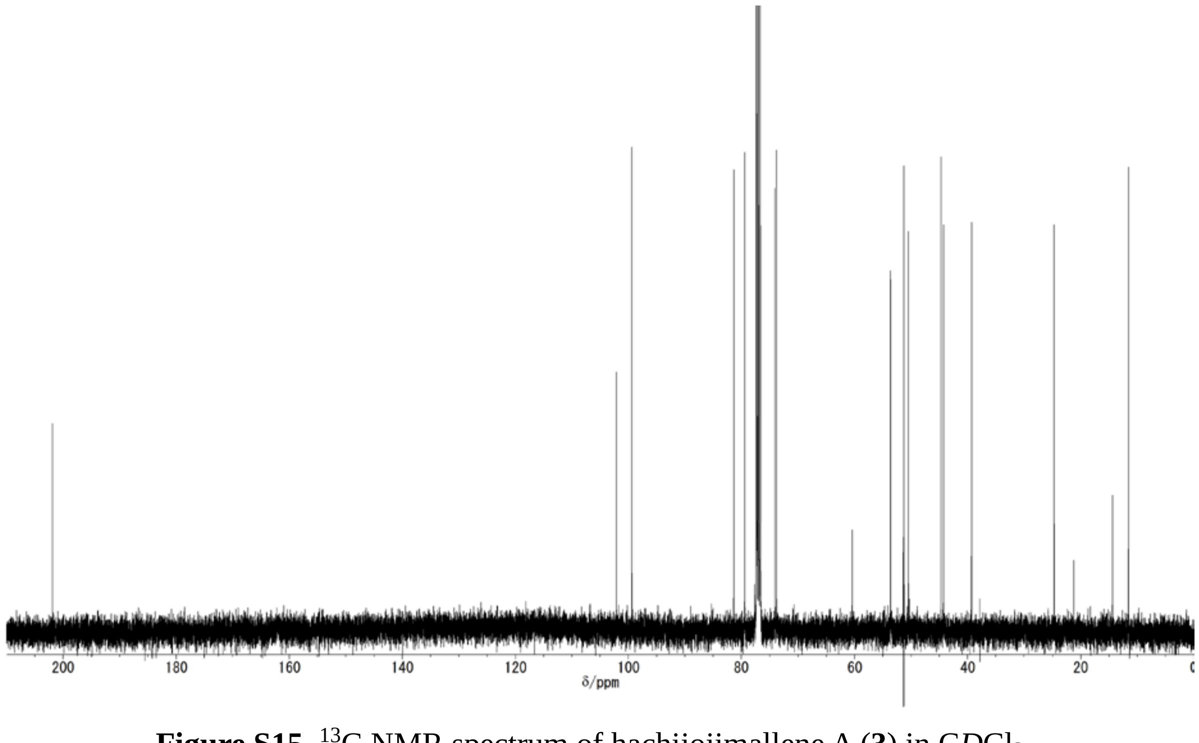

Figure S15. ^{13}C NMR spectrum of hachijojimallene A (**3**) in C*D*Cl$_3$.

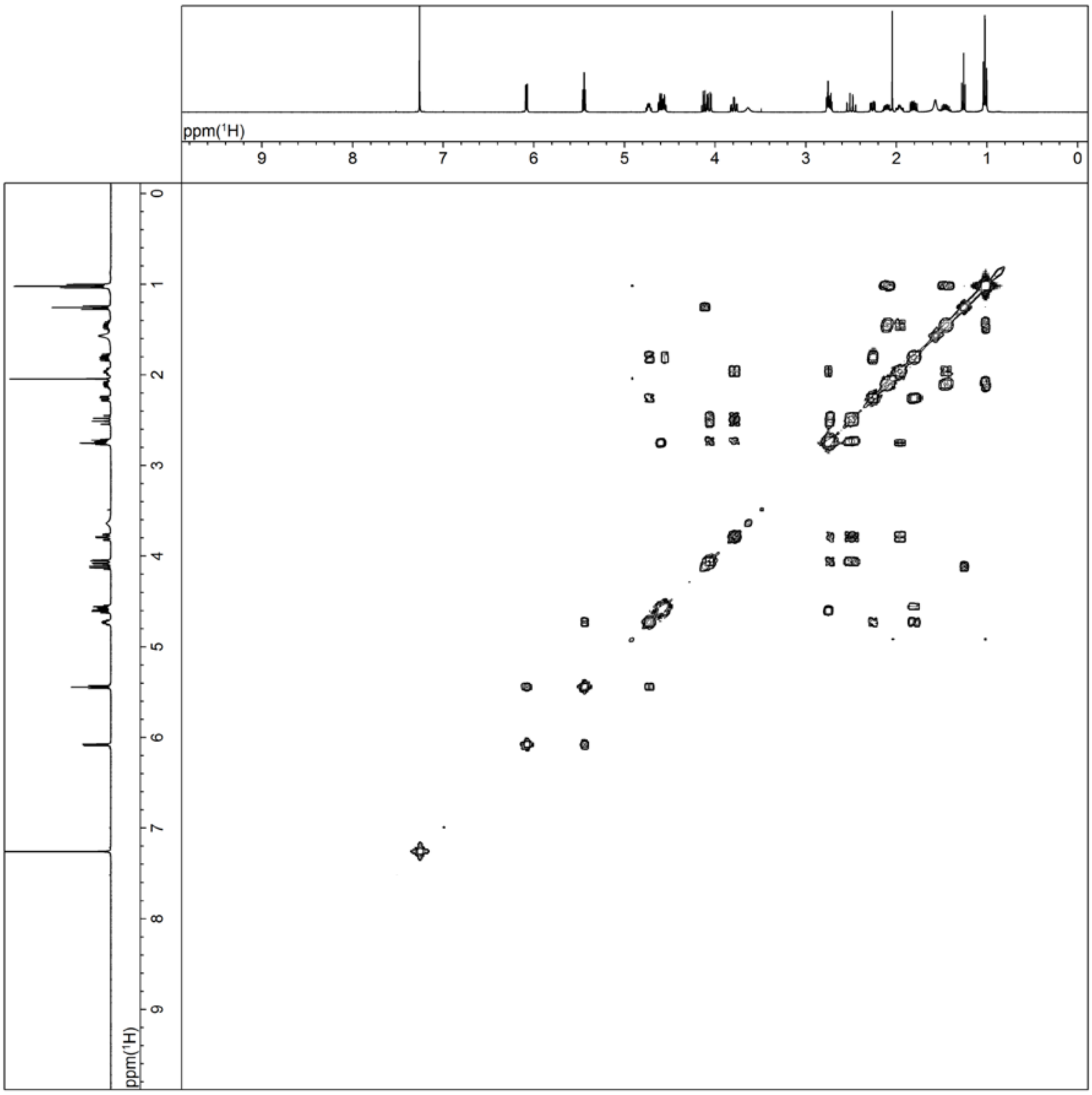

Figure S16. ^1H–^1H COSY spectrum of hachijojimallene A (**3**) in C*D*Cl_3.

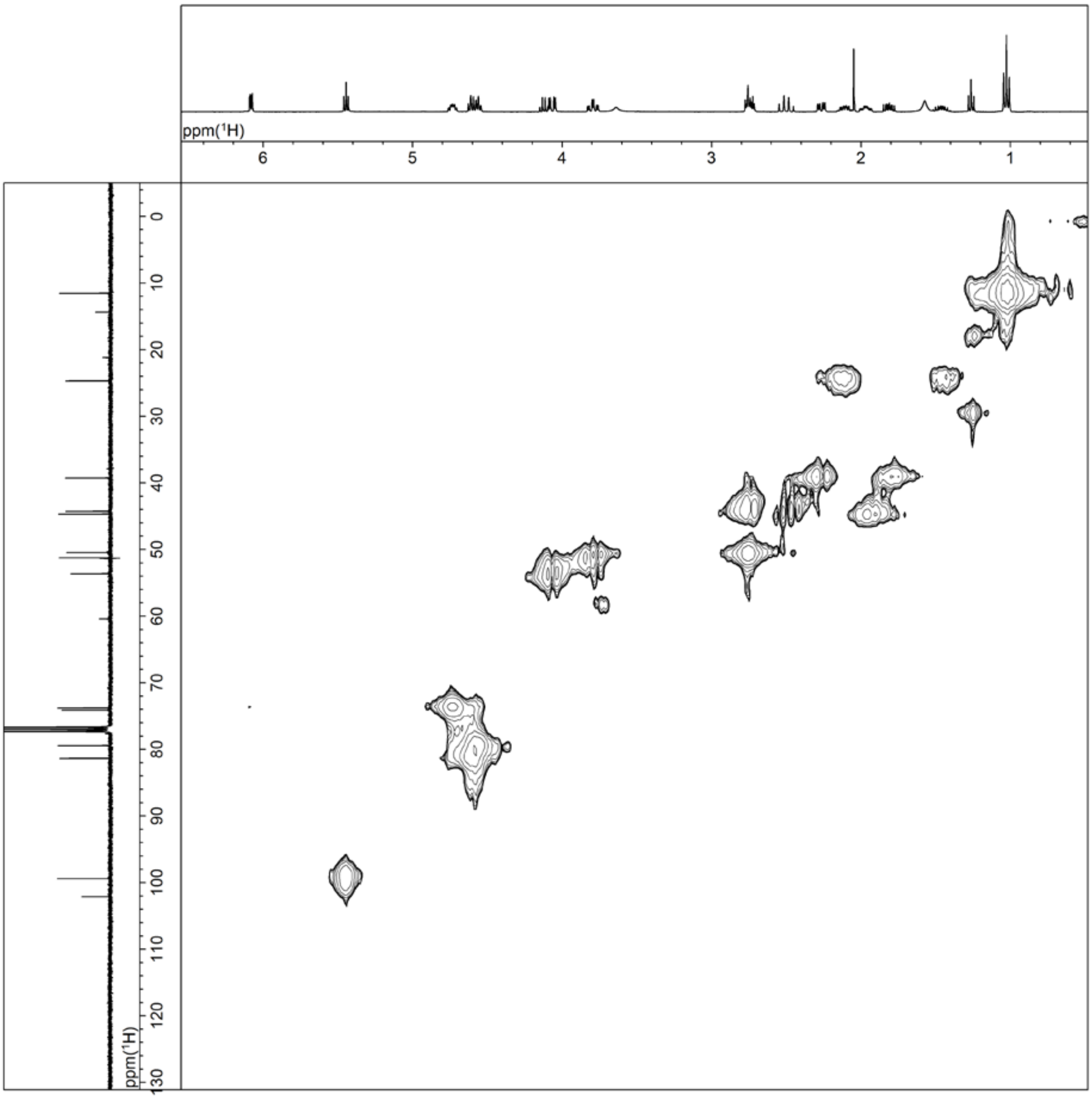

Figure S17. HMQC spectrum of hachijojimallene A (**3**) in C*D*Cl$_3$.

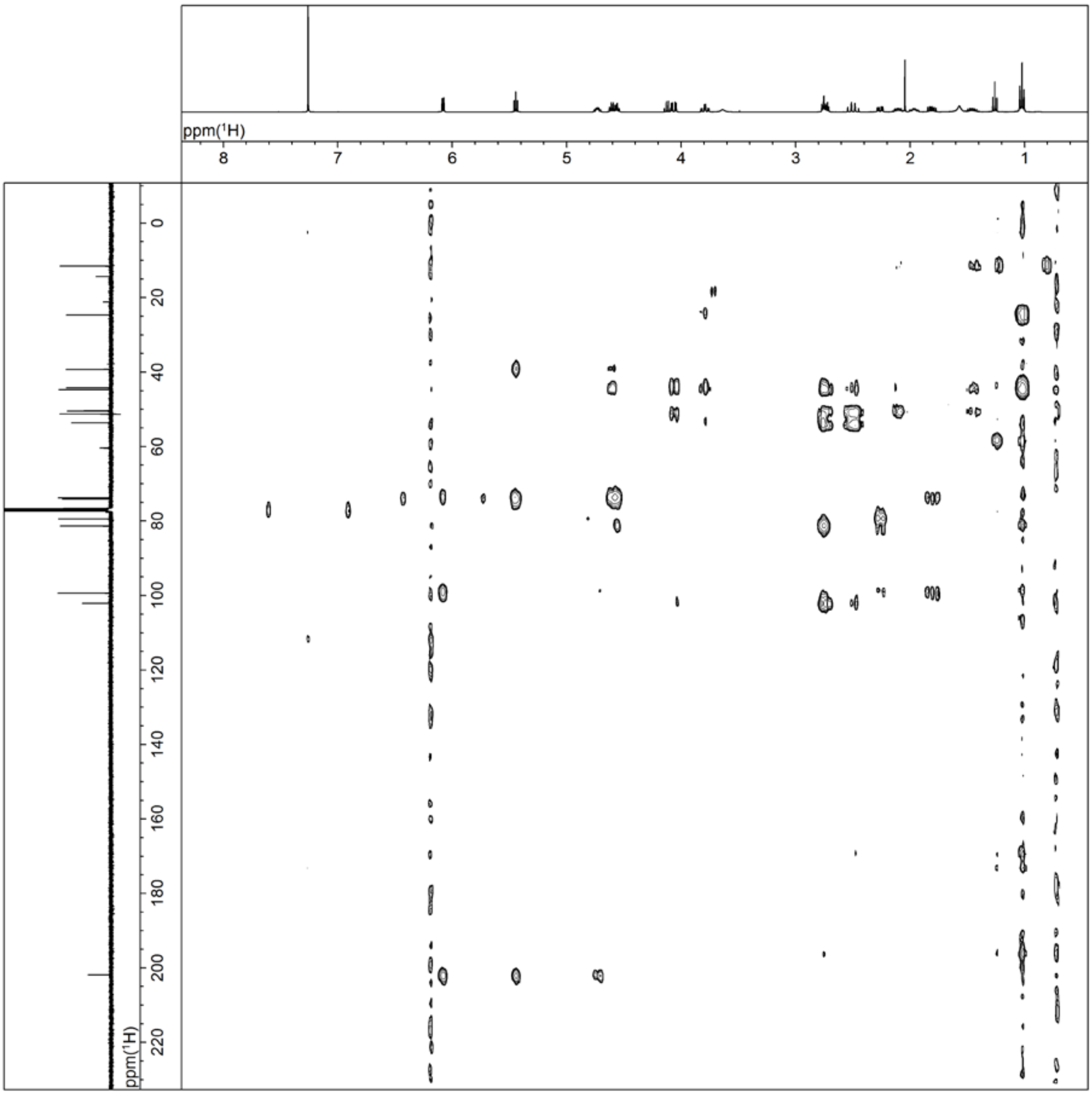

Figure S18. HMBC spectrum of hachijojimallene A (**3**) in C*D*Cl$_3$.

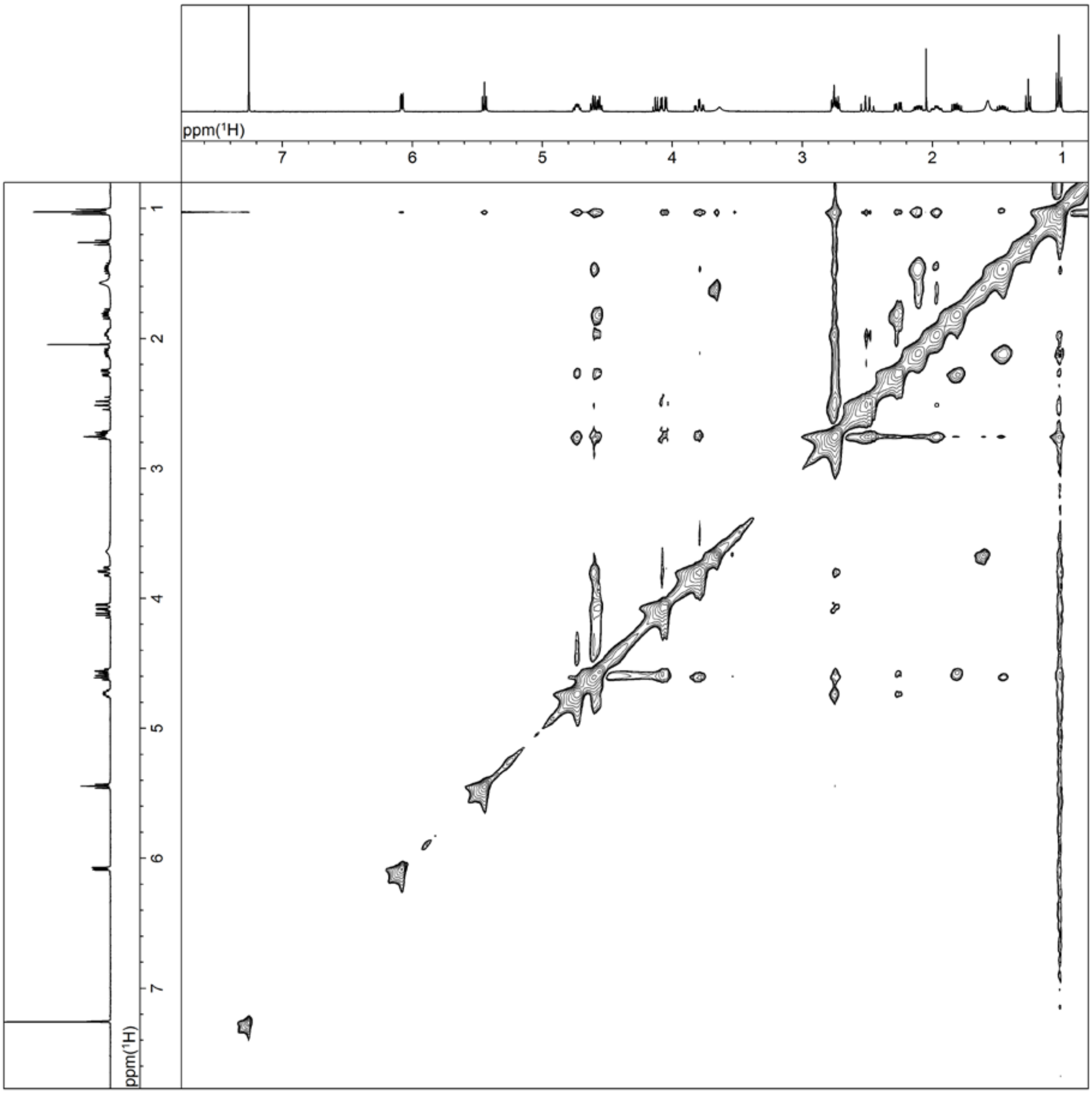

Figure S19. NOESY spectrum of hachijojimallene A (**3**) in C*D*Cl$_3$.

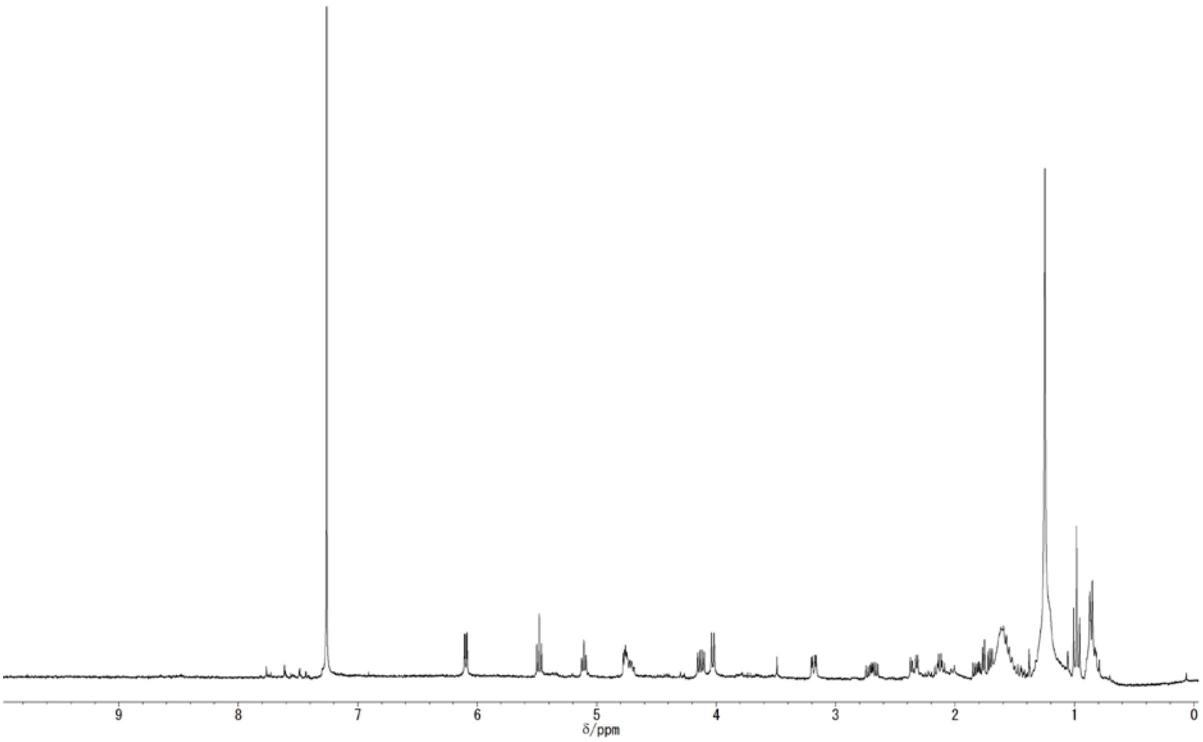

Figure S20. ^{1}H NMR spectrum of hachijojimallene B (**4**) in C*D*Cl_3.

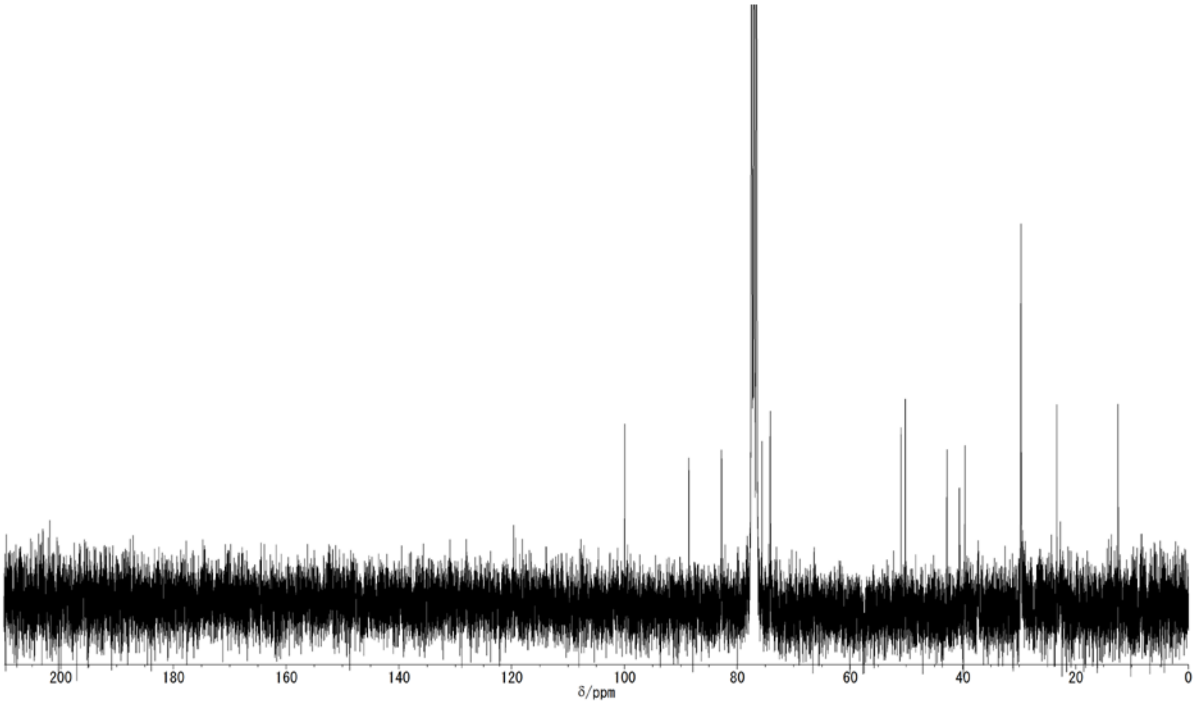

Figure S21. ^{13}C NMR spectrum of hachijojimallene B (**4**) in C*D*Cl_3.

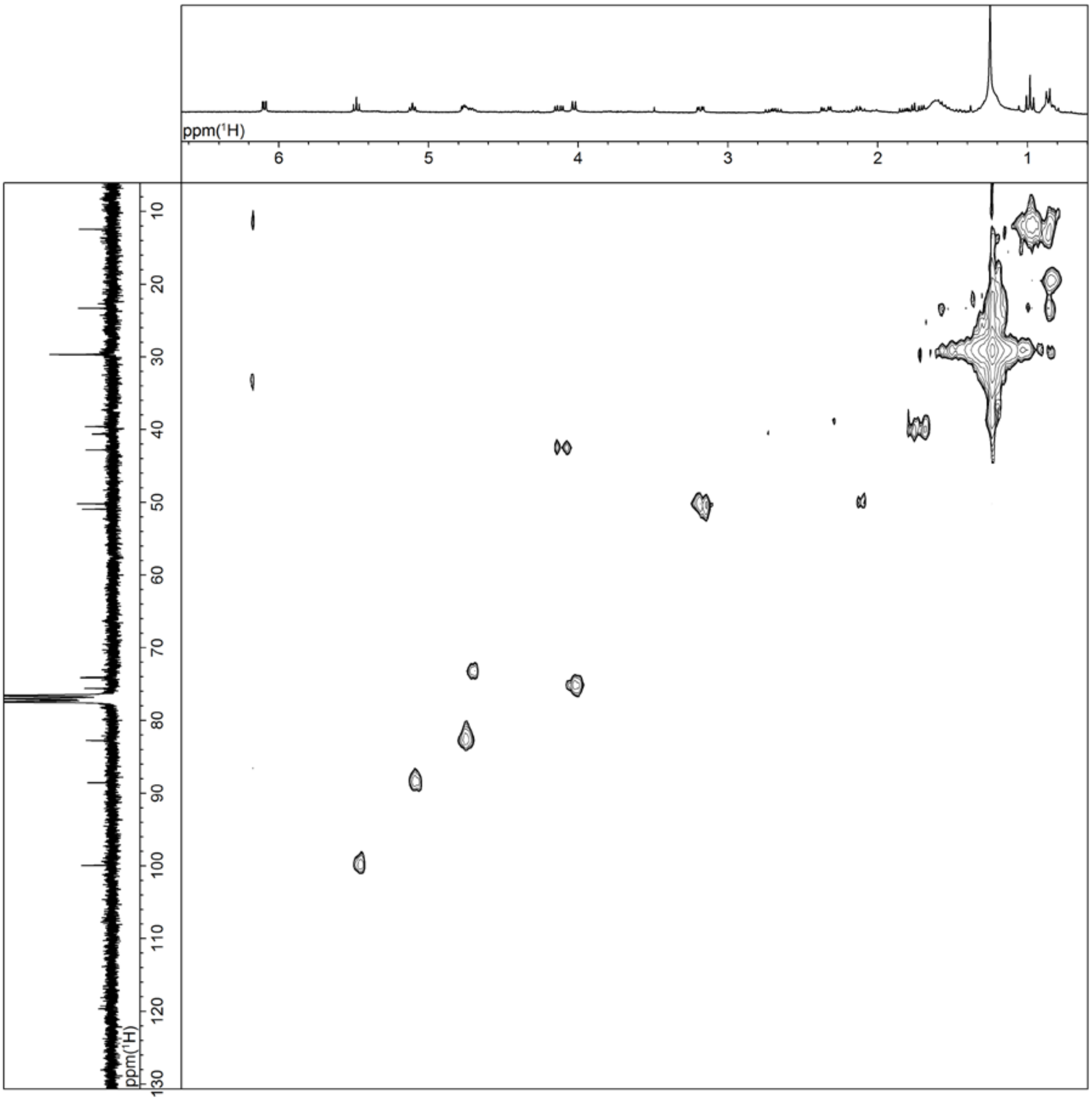

Figure S22. HMQC spectrum of hachijojimallene B (**4**) in C*D*Cl_3.

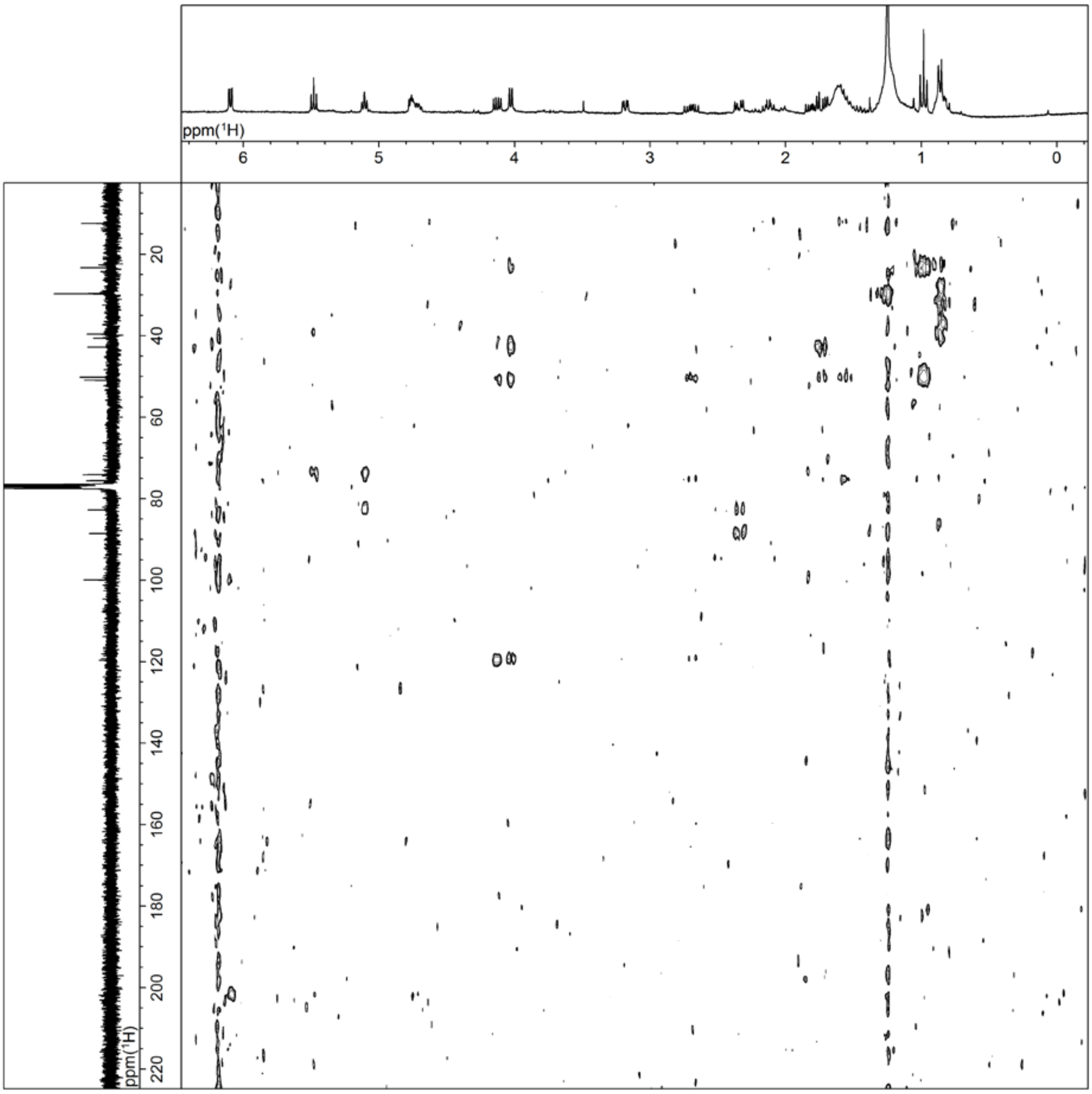

Figure S23. HMBC spectrum of hachijojimallene B (**4**) in C*D*Cl$_3$.

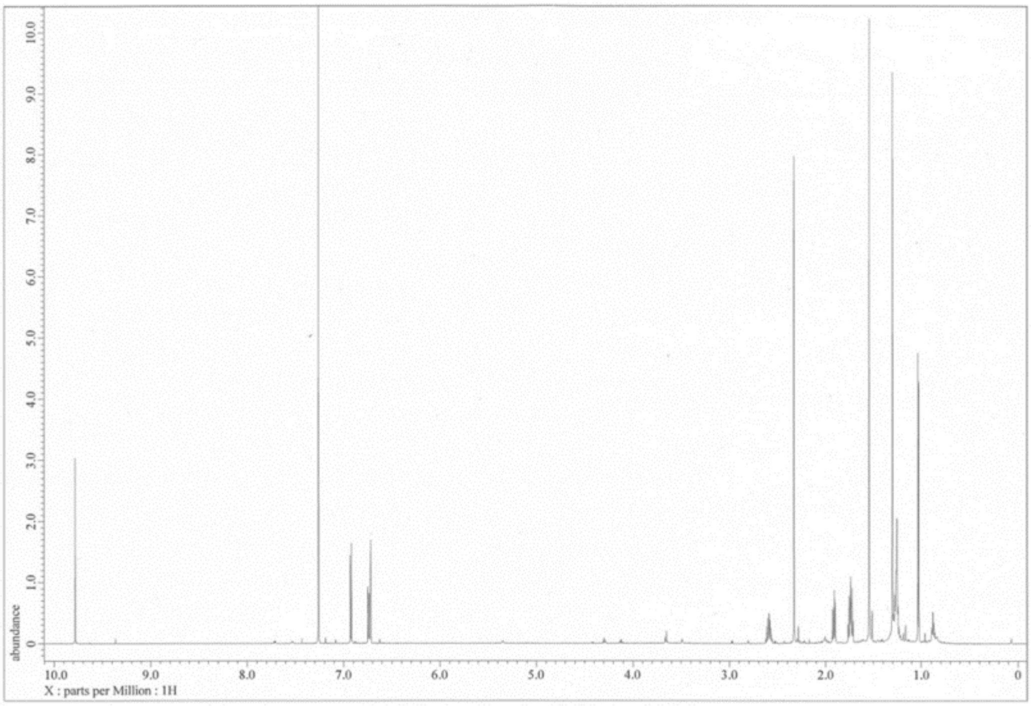

Figure S24. ^{1}H NMR spectrum of debromoaplysinal (**5**) in C*D*Cl_3.

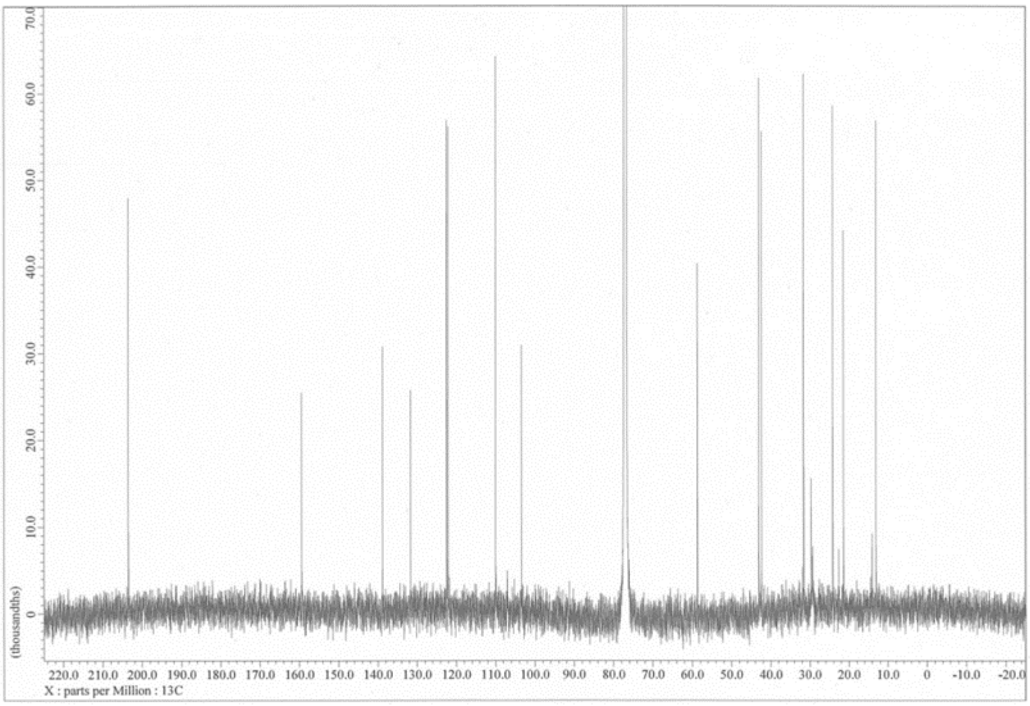

Figure S25. ^{13}C NMR spectrum of debromoaplysinal (**5**) in C*D*Cl_3.

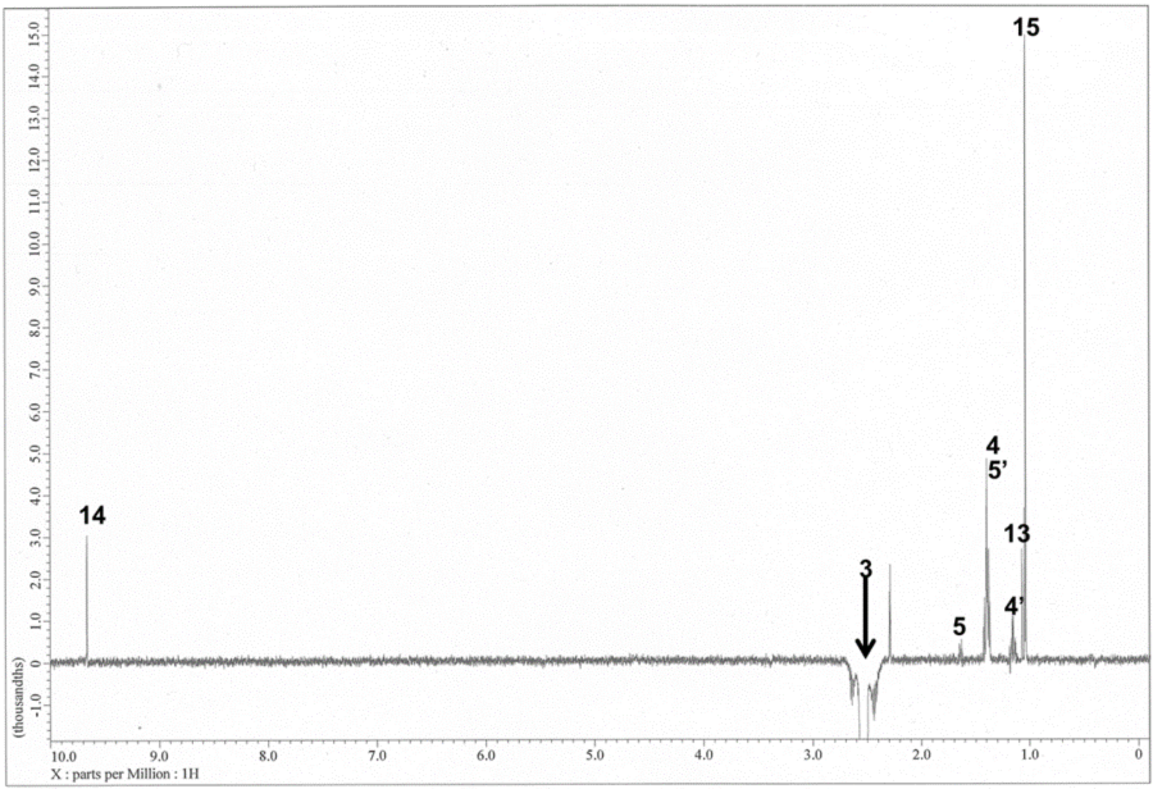

Figure S26. DPFGSE 1D NOE spectrum of debromoaplysinal (**5**) in C*D*Cl_3.

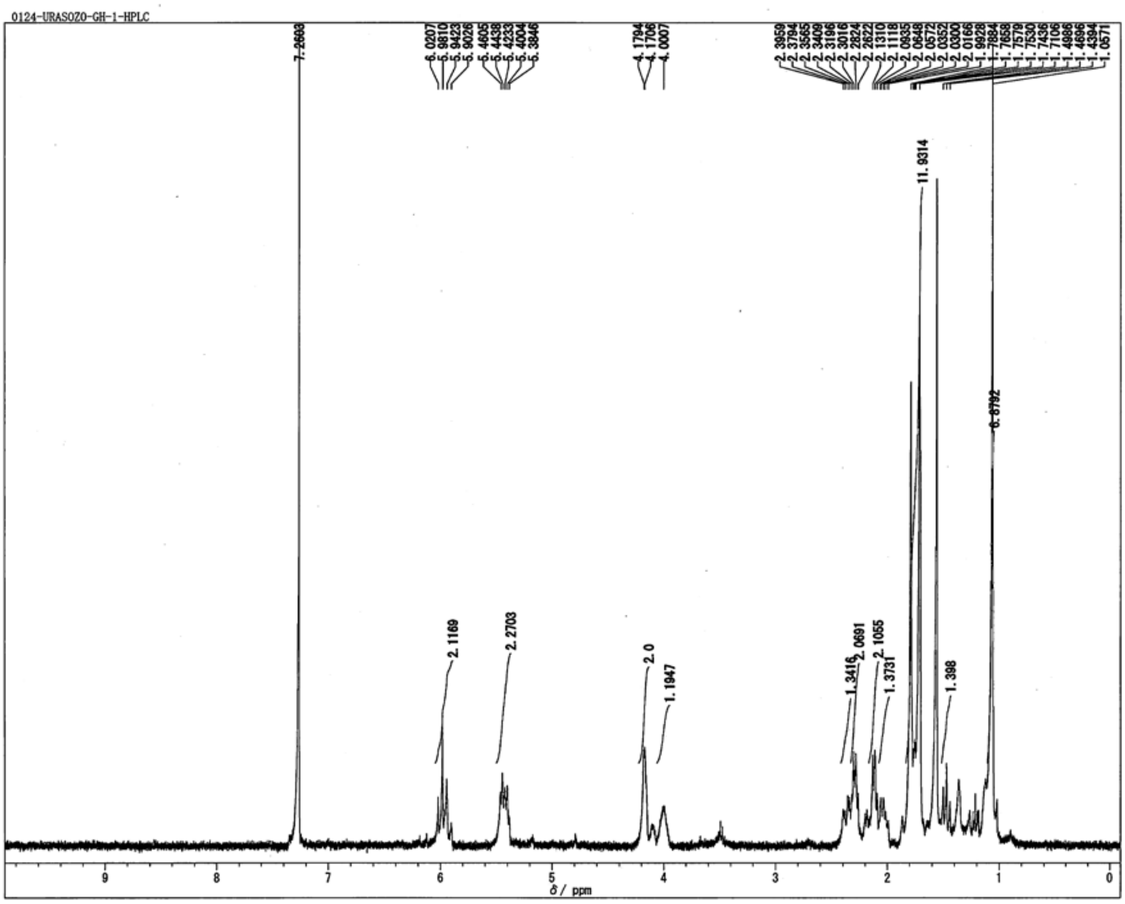

Figure S27. ^{1}H NMR spectrum of 11,12-dihydro-3-hydroxyretinol (**6**) in C*D*Cl$_3$.

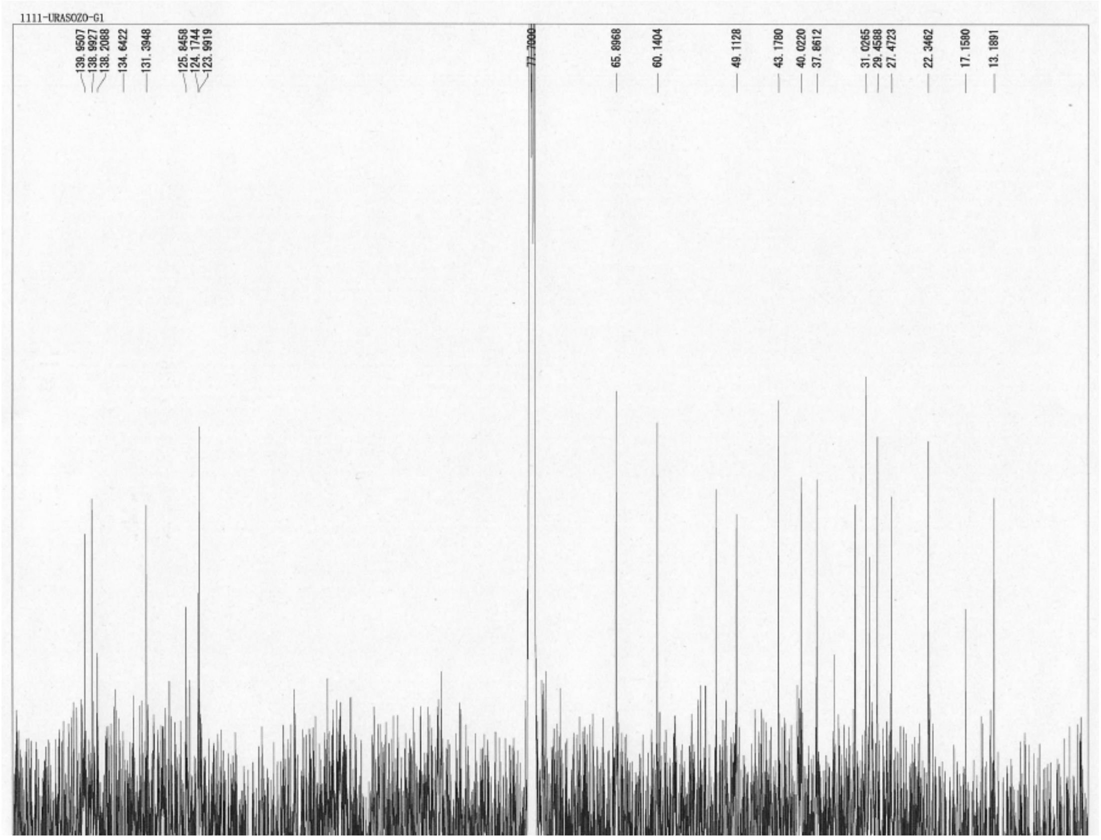

Figure S28. ^{13}C NMR spectrum of 11,12-dihydro-3-hydroxyretinol (**6**) in C*D*Cl$_3$.
